# Spontaneous mentalizing in patients with schizophrenia spectrum disorders: a meta-analysis

**DOI:** 10.1017/S0033291725100755

**Published:** 2025-07-16

**Authors:** András Hajnal, Tímea Csulak, Dóra Hebling, Kornélia Farkas Borbásné, Márton Herold, Gergő Berke, Zoltán Sipos, Borbála Pethő, Eszter Varga, Tamás Tényi, Péter Mátrai, Péter Hegyi, Noémi Albert, Róbert Herold

**Affiliations:** 1 Department of Psychiatry and Psychotherapy, Medical School, University of Pécs, Pécs, Hungary; 2 Institute for Translational Medicine, Medical School, University of Pécs, Pécs, Hungary; 3 Department of Pediatrics, Medical School, University of Pécs, Pécs, Hungary

**Keywords:** implicit, mentalization, mentalizing, neurocognition, schizophrenia, spontaneous, theory of mind

## Abstract

**Background:**

Spontaneous mentalizing refers to the capacity to attribute mental states to oneself and others without explicit prompts or conscious deliberation. This process enables individuals to comprehend and anticipate social behaviors in a more intuitive manner. Individuals diagnosed with schizophrenia frequently demonstrate deficits in this domain, which contribute to impaired social functioning. The present meta-analysis aims to assess the extent of spontaneous mentalizing impairments in schizophrenia.

**Methods:**

A comprehensive search was conducted in four prominent databases: MEDLINE, EMBASE, CENTRAL, and Web of Science. Following the review of the retrieved records and subsequent citation searching, a total of 15 studies were selected for inclusion in the quantitative synthesis. The data of 526 patients diagnosed with schizophrenia and 536 controls were subjected to analysis. Effect sizes for intentionality and appropriateness were computed utilizing weighted or standardized mean differences, and heterogeneity was evaluated.

**Results:**

Patients with schizophrenia exhibited substantial impairments in intentionality and appropriateness during mentalizing tasks, with large effect sizes. No significant differences were observed in random movement tasks, although patients also demonstrated deficits in interpreting goal-directed movements. Furthermore, high heterogeneity in some outcomes and variability in study methodologies were also noted.

**Conclusions:**

This analysis corroborates substantial spontaneous mentalizing deficits in schizophrenia, underscoring their potential role in impaired social functioning. In conjunction with previous analyses, the present findings emphasize the pervasive nature of mentalizing deficits in schizophrenia, encompassing explicit, implicit, and spontaneous dimensions. These results hold significant implications for therapeutic strategies designed to augment social cognition in individuals with schizophrenia.

## Background

To establish and maintain everyday interpersonal relationships, individuals must accurately represent their own and others’ mental states, encompassing intentions and beliefs. These intricate processes are collectively referred to as mentalizing or theory of mind (ToM), enabling individuals to comprehend and anticipate the behaviour of others.

However, mentalizing is a multifaceted ability comprising various subcomponents, such as detecting intentions and reasoning about mental states. Furthermore, it relies on other cognitive processes, including emotion recognition and social knowledge. Recent theories emphasize that mentalizing encompasses both declarative and procedural processes (Duclos, Desgranges, Eustache, & Laisney, 2018). Consequently, mentalizing is hypothesized to possess both explicit and implicit aspects (Apperly & Butterfill, 2009; Butterfill & Apperly, 2013). Implicit mentalizing is believed to be present during the early, preverbal period, suggesting it is a fundamentally biologically rooted skill (Heyes & Frith, 2014). It operates autonomously, reflexively, fast, and unconsciously, independent of verbal abilities. In contrast, the explicit dimension is slower but reflective and conscious, primarily relying on verbal skills. Explicit mentalizing emerges as a result of cultural learning processes (Heyes & Frith, 2014).

The term ‘spontaneous’ is frequently employed as a synonym for ‘implicit’ in mentalizing research (Csulak & Herold, 2021). However, Senju (2013) posits that spontaneous mentalizing differs from implicit mentalizing in several ways. Unlike implicit mentalizing, spontaneous mentalizing is not as obligatory as automatic processing. It is not necessarily unconscious and can interfere with competing tasks. Additionally, spontaneous mentalizing is conceptualized as an immediate, real-time reasoning ability that requires a rapid decoding of biological motion and action. It also serves as a prerequisite for explicit and verbal ToM activity (Koelkebeck et al., 2013). Consequently, spontaneous mentalizing reflects the preparedness of the mentalizing capacity for recruitment. Although implicit, spontaneous, and explicit mentalization are complex and interconnected in nature, it is crucial to note that the measurement paradigms differ significantly in all three cases. Measures of explicit mentalizing require conscious recognition and description of another’s mental state. Implicit mentalizing tasks use indirect approaches, such as nonprompted behaviors. Indirect performance indicators include reaction time without verbal responses. Spontaneous mentalizing is assessed through tasks that do not explicitly instruct interpreting mental states, but it is measured by the spontaneous use of mental state terms.

Several meta-analyses have confirmed the presence of mentalizing deficits in schizophrenia (Bora & Pantelis, 2013, 2016; Bora, Yucel, & Pantelis, 2009; Sprong et al., 2007). This impairment is observed both during the acute phase (Bora et al., 2009; Martin et al., 2014) and during remission (Fekete et al., 2020; Herold, Tényi, Lénárd, & Trixler, 2002). Studies on the correlation between symptomatology and ToM show heterogeneity, with no clear link between positive, negative, or disorganization/cognitive symptom dimensions. A recent meta-analysis found a small to moderate association between cognitive/disorganization symptoms and negative symptoms but a weaker association with positive symptoms (Thibaudeau et al., 2023). ToM is generally linked to neurocognitive function, with moderate correlations between ToM and executive functions, memory, attention, and other cognitive domains (Thibaudeau et al., 2020). However, ToM often emerges independently of these functions, suggesting a distinct social-cognitive impairment (Parola et al., 2020). Furthermore, it can be detected in the asymptomatic first-degree relatives of patients (Bora & Pantelis, 2013; Herold et al., 2018; Janssen, Krabbendam, Jolles, & Van Os, 2003). Individuals clinically high-risk for psychosis and those with first-episode psychosis also exhibit lower mentalizing performance (Bora & Pantelis, 2013). Longitudinal studies have demonstrated a deterioration in social functionality in schizophrenia (Velthorst et al., 2017). Mentalizing emerged as a robust predictor of functional outcomes, demonstrating the strongest associations with productive activities, such as vocational and educational pursuits, and performance-based functional measures. (Fett et al., 2011; Thibaudeau, Cellard, Turcotte, & Achim, 2021).

The majority of studies examined the explicit abilities of individuals with schizophrenia, and most of these studies have revealed a marked decrease in explicit mentalizing functions (Csulak et al., 2022). However, increasingly more studies are focusing on the involvement of implicit and spontaneous mentalizing in schizophrenia. In a previous article, we summarized the results of implicit mentalizing research (Csulak et al., 2022). We demonstrated in a meta-analysis that implicit mentalizing is also impaired in schizophrenia but not to the same extent as explicit abilities. Furthermore, neurocognitive deficits may significantly limit the efficiency of implicit skills. In our meta-analysis, we focused on those studies that measure implicit mentalizing with automatic behavioural signs without verbal responses.

The primary objective of this meta-analysis is to investigate the nature of spontaneous mentalizing performance in individuals diagnosed with schizophrenia. Some researchers argue that explicit tasks may not accurately reflect everyday social interactions because they are inherently problem-solving in nature (Klin, 2000). Furthermore, task performance can also be influenced by neurocognitive abilities (Thibaudeau et al., 2020). Conversely, paradigms employing indirect instructions have been demonstrated to effectively identify impairment in patients, even when executive function is not compromised (Langdon, Connors, & Connaughton, 2017). The tasks designed to assess spontaneous mentalization necessitate the unprovoked activation of mentalizing abilities. Several paradigms have been developed to measure mentalizing abilities indirectly through indirect instructions (see Appendix for detailed description). Perhaps the most widely used test is the animated triangle task (ATT) (Abell, Happe, & Frith, 2000) It contains animations of two triangles performing three types of movements: random (movements without purpose), goal-directed (GD), and scripts involving mentalizing interactions (i.e., coaxing, mocking). Random and GD animations serve as control tasks. The performances in each animation are scored based on intentionality (the extent to which mental state attribution is expressed in describing the interactions) and appropriateness (the degree to which the description accurately reflects the intended meaning). Intentionality performance is particularly well-suited to assess mentalizing abilities (Bliksted, Ubukata, & Koelkebeck, 2016). An earlier meta-analysis of studies employing ATT revealed significantly lower intentionality and appropriateness scores, accompanied by substantial effect sizes, in individuals with schizophrenia (Bliksted et al., 2016). In addition to ATT, other paradigms have also been developed. The modified versions of Heider and Simmel’s (Heider & Simmel, 1944) animation are also employed in schizophrenia research (Bell, Fiszdon, Greig, & Wexler, 2010; Langdon, Connors et al., 2017). The Social Attribution Task – Multiple Choice version (SAT-MC) is based on this animation and was designed to reduce the reliance on verbal function with multiple-choice questions after the Heider and Simmel animation (Bell et al., 2010). The joke appreciation task utilizes cartoons, where the comprehension of a joke hinges on spontaneously recognizing false beliefs (Langdon, Flynn, Connaughton, & Brüne, 2017). The majority of studies conducted with individuals diagnosed with schizophrenia have indicated that patients employ less appropriate social terms. Consequently, it is plausible that they exhibit reduced receptivity to various cues that trigger spontaneous mentalizing (Langdon, Connors et al., 2017).

In addition to our meta-analysis on implicit mentalizing, we consider it imperative to summarize the findings on spontaneous mentalizing as well. The primary objective of the present study was to assess the extent of spontaneous mentalizing performance in schizophrenia through a meta-analysis. We postulated that individuals with schizophrenia would exhibit impairments in tasks evaluating spontaneous mentalizing but not in control conditions assessing GD or random interactions. To achieve this, we included case–control studies employing indirect instructions that measure spontaneous verbal descriptions of social interactions.

## Methods

This meta-analysis adhered to the Preferred Reporting Items for Systematic Reviews and Meta-Analyses (PRISMA) guidelines (Page et al., 2021). The review protocol was registered on PROSPERO (CRD42022318909), and no protocol deviations were observed.

### Search strategy

A comprehensive systematic search was conducted in four prominent databases: MEDLINE, EMBASE, Cochrane Central Register of Controlled Trials (CENTRAL), and Web of Science. The search date was March 8, 2022. The search query employed the following keywords: ((implicit) OR (spontaneous)) AND ((theory of mind) OR (mentalizing) OR (mentalization)) AND (schizophrenia). The search encompassed all fields and textual content within each database. No restrictions or filters were applied to the search.

### Selection and eligibility criteria

The search results were synthesized using reference manager software (EndNoteX9; Clarivate Analytics, Philadelphia, Pennsylvania). After both automatic and manual duplicate removal, a screening process was conducted based on title, abstract, and full text. Subsequently, the references and citations of the full-text screening records were reviewed. The selection process was conducted by two independent researchers (AH and TC). Disagreements were resolved by an independent third investigator (RH). Reference lists and publication citations (Google Scholar search engine) of the included studies were screened to identify additional studies.

We included studies examining spontaneous mentalization in patients with schizophrenia (and patients with schizoaffective and schizophreniform disorders). Studies from which we could not extract data of sufficient quality were excluded. Articles concerning patients with any other major psychiatric disorders (e.g., bipolar affective disorder) have been excluded.

### Data extraction

From each of the eligible studies, the following data was extracted: first author, publication year, study design, country, number of centers, studied population, gender distribution, age distribution, number of patients, accuracy, intentionality (during random, GD, and mentalizing tasks separately), and rate of mentalizing terms during mentalizing tasks. Data were extracted by two independent review authors. Disagreements were resolved by an independent third investigator.

### Risk of bias assessment

The ‘Quality In Prognosis Studies’ (QUIPS) methodology was employed, as per the recommendations of The Cochrane Prognosis Methods Group (PMG). Two researchers were involved in the assessment process. Any disagreements were resolved through a third review author (Supplementary Figures 1–7).

### Statistical analysis

For continuous outcomes, weighted mean differences (WMDs) or when different scoring scales or paradigms had been employed to measure the outcomes, and standardized mean differences (SMD) were computed with 95% confidence intervals.

In terms of appropriateness, SMD was calculated for both mentalizing and control tasks, as the studies employed distinct tasks (ATT, SAT-MC, joke appreciation). Additionally, SMD was computed for GD tasks, given the utilization of varying scoring scales. Given that several studies utilized the same paradigm (ATT) and scoring scale, we also calculated WMD within appropriateness. ATT is the sole paradigm for which intentionality was additionally measured, necessitating the calculation of WMD for this outcome.

We applied the Hedges method to estimate the SMD since a few studies had sample sizes less than 20. Random effect models were utilized to pool the effect sizes. A *p*-value less than 0.05 was deemed statistically significant. Additionally, we applied the restricted maximum likelihood estimator and the Knapp–Hartung adjustments (Knapp & Hartung, 2003) to calculate the heterogeneity variance *τ*^2^ and the confidence interval of the overall effect, respectively. The results of the meta-analyses were presented in forest plots.

Heterogeneity was assessed using the I^2^ statistics. Following the guidelines provided in the Cochrane Handbook (Chandler et al., 2019), I^2^ values were categorized as follows: 0%–40%, 30%–60%, 50%–90%, and 75%–100%. These categories correspond to the following interpretations: ‘Might not be important,’ ‘Moderate,’ ‘Substantial,’ and ‘Considerable,’ respectively. Heterogeneity was considered significant when the *p*-value was less than 0.1. For outcomes involving at least 10 studies, we conducted a funnel plot analysis and performed Egger’s test to assess potential publication bias. All analyses were conducted using the R environment (R Core Team, 2021; R: A language and environment for statistical computing. R Foundation for Statistical Computing, Vienna, Austria. R version 4.1.2, 2021-11-01).

Furthermore, we investigated the association between intentionality and appropriateness score during mentalizing tasks and mean age and mean years of education for both the schizophrenia and control groups. This was achieved by fitting a linear meta-regression model. (Supplementary Figures 14–21).

## Results

### Systematic search and selection

**Figure 1.**
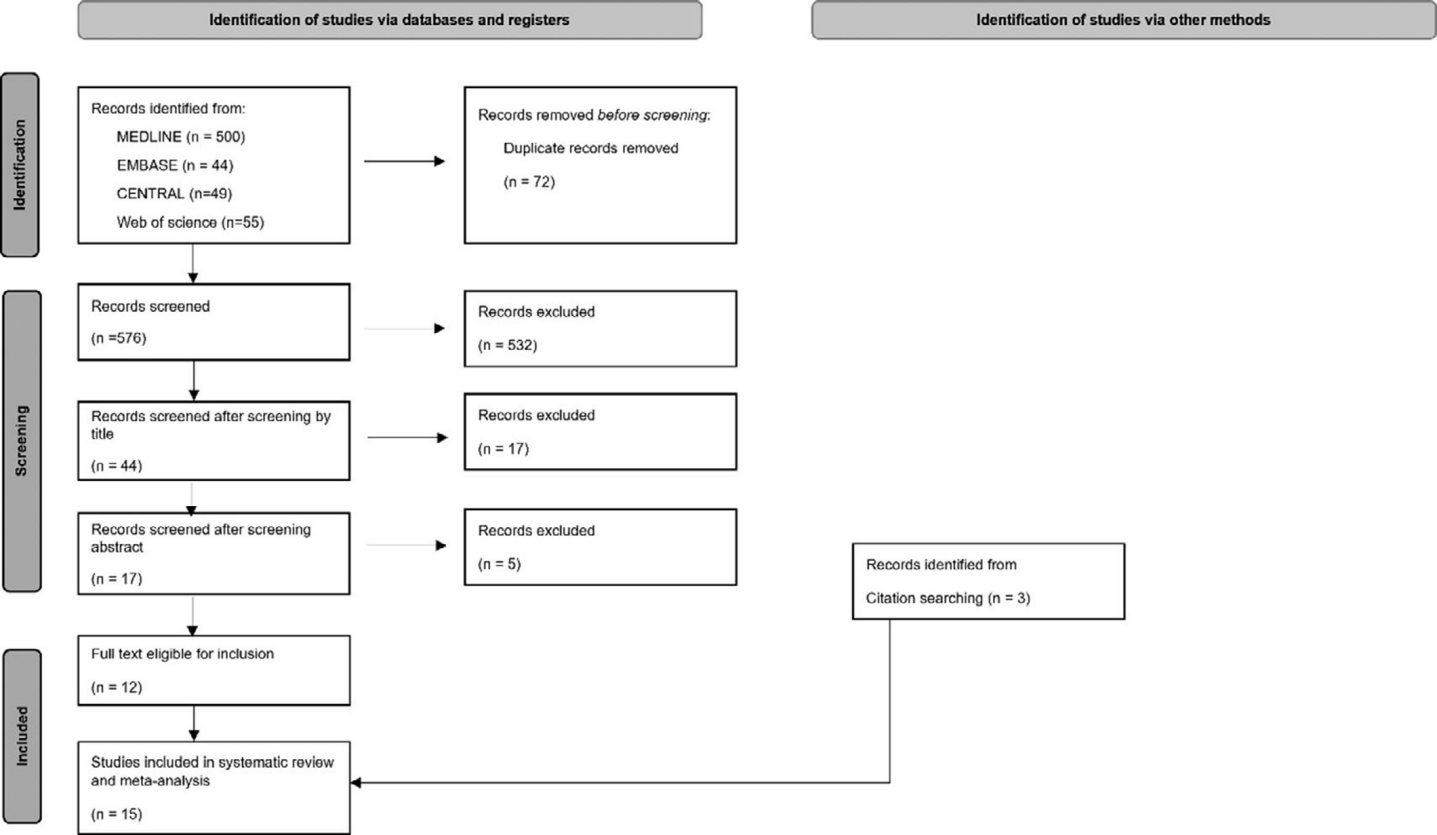
PRISMA flowchart for the study selection process (Page et al., 2021).

The systematic search yielded 648 records. After the automatic and manual removal of duplicates, 576 records remained. The flowchart of the publication selection process is presented in Figure 1. After checking the records and conducting citation searching, 15 studies were included in the quantitative synthesis (Bell et al., 2010; Beck et al., 2020; Bliksted et al., 2014, 2019; Das et al., 2012; Horan et al., 2009; Koelkebeck et al., 2013; Langdon & Ward, 2009; Langdon, Flynn et al., 2017; Langdon, Boulton, Connaughton, & Gao, 2020; Lee et al., 2018; Lugnegård, Hallerbäck, Hjärthag, & Gillberg, 2013; Pedersen et al., 2012; Roux, Smith, Passerieux, & Ramus, 2016; Veddum, Pedersen, Landert, & Bliksted, 2019). The meta-analysis included 526 patients and 536 controls in its analysis. The characteristics of the included studies are presented in Table 1.

**Table 1.** The characteristics of the included studies

	Task design	No. of patients (female of total %)	Age of patients (mean + SD)	No. of controls (female of total %)	Age of controls (mean + SD)	Medication (antipsychotic)
Beck et al. (2020) (Chinese)	ATT	35 (54%)	28.46 (25.56; 31.35)	38 (46%)	30.32 (27.54; 33.09)	All but two
Beck et al. (2020) (Danish)	ATT	31 (42%)	25.10 (23.63; 26.56)	29 (45%)	24.38. (22.64; 26.12)	All but seven
Bell et al. (2010)	SAT-MC	66 (39.4%)	42.73 (10.4)	85 (88%)	31.72 (8.58)	All but nine
Bliksted et al. (2014)	ATT	36 (47.2%)	22.7 (21.6; 23.7)	36 (47.2%)	22.7 (21.6; 23.7)	All but one
Bliksted et al. (2019)	ATT	17 (29.41%)	23.94 (21.76; 26.11)	17 (29.41%)	23.59 (21.50; 25.68)	All but nine
Das et al. (2012)	ATT	23 (0%)	34.5 (8.4)	22 (0%)	33.5 (8.4)	All but one
Horan et al. (2009)	ATT	55 (24%)	40.1 (10.8)	44 (24%)	39.7 (9.1)	All
Koelkebeck et al. (2013)	ATT	18 (33.33%)	34.9 (10.1)	30 (53.33%)	39.1 (10.8)	All
Langdon and Ward (2009)	Joke appreciation task	30 (36.67%)	38.5 (1.8)	26 (38.46%)	35.3 (2.6)	All but one
Langdon, Connors, and Connaughton (2017)	Joke appreciation	50 (42%)	42.4 (10.7)	30 (56.67%)	40.5 (13.9)	–
Langdon et al. (2020)	ATT /joke appreciation	23 (30.43%)	45.8 (8.7)	20 (30%)	46.9 (12.7)	All
Lee et al. (2018)	SAT-MC	60 (49.20%)		60 (51.67%)		–
Lugnegard et al. (2013)	ATT	36 (38.89%)	28.8 (4.1)	50 (62%)	28.8 (9.3)	All but six
Pedersen et al. (2012)	ATT	15 (40%)	29.0 (8.2)	14 (55.5%)	29.9 (8.8)	All
Roux et al. (2016)	ATT	29 (27.59%)	39 (12.5)	29 (34.48%)	40.7 (13.5)	All
Veddum et al. (2019)	ATT	21 (52.4%)	40.48 (34.84; 46.11)	21 (52.4%)	25.45 (21.30; 29.61)	–

### Intentionality

For the purpose of investigating intentionality during ToM animations, data from ten studies (all employing the ATT) were analysed (Supplementary Figure 8). These studies involved a total of 269 patients and 282 controls. A statistically significant difference was observed between the two groups, with a *p*-value less than 0.001. The mean difference (MD) was −0.99, and the confidence interval (CI) ranged from −1.39 to −0.59. The between-study heterogeneity, expressed as I^2^ value, was 88.2%, indicating substantial heterogeneity.

**Figure 2.**
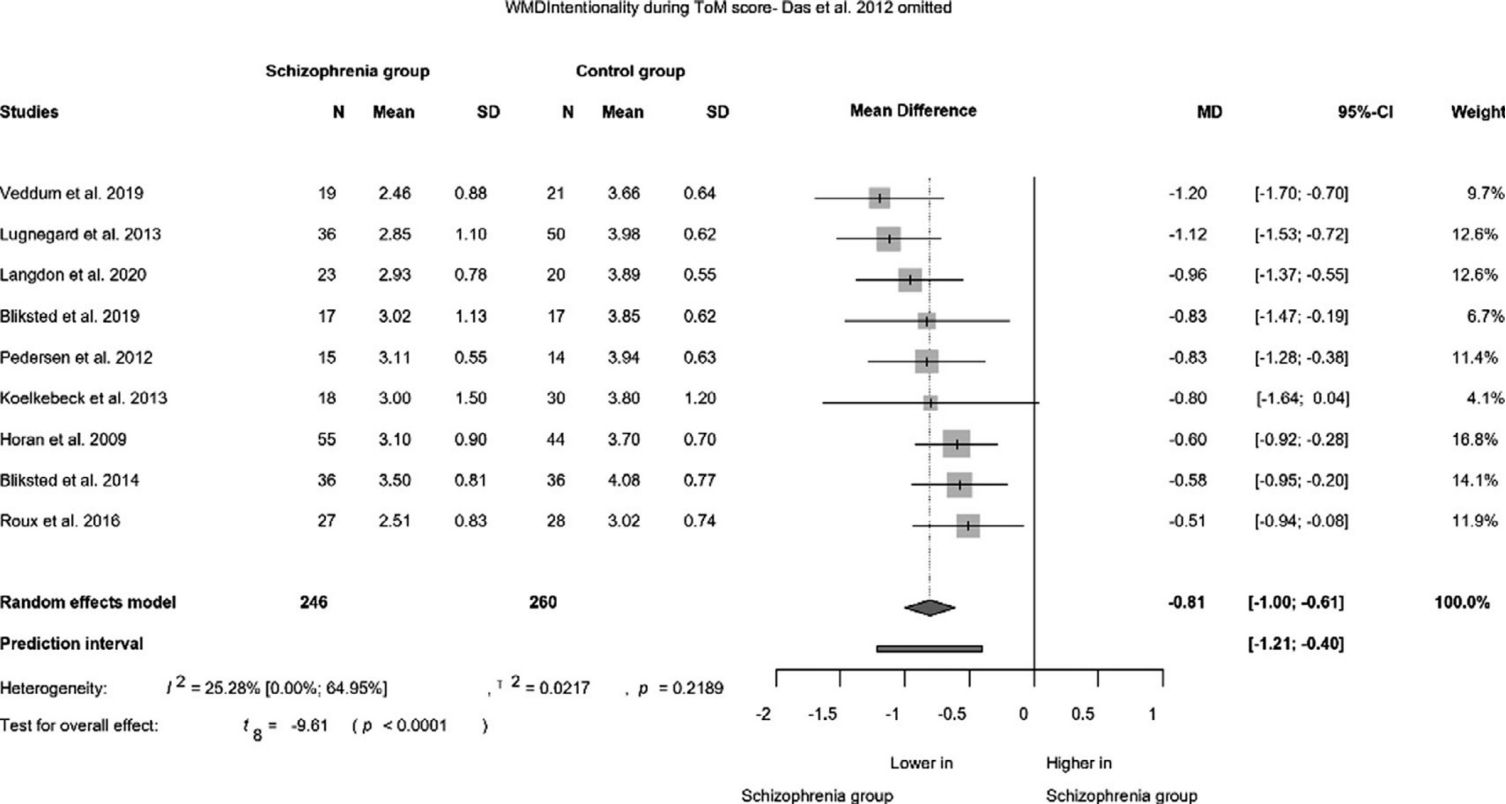
Intentionality Score during ToM tasks (Omitted by Das et al., 2012).

Given the divergent findings of the Das et al. study compared to the others, we conducted a calculation by excluding this data (Figure 2). Consequently, the distinction between the two groups remains statistically significant (MD: −0.81; CI (−1.00; −0.61), *p* < 0.001), albeit with a substantial reduction in heterogeneity (*I*^2^ = 25.28%).

A total of eight studies were selected for analyses of intentionality during random tasks, involving 231 patients and 246 controls (Supplementary Figure 9). The mean difference (pooled effect size) between the two groups was 0.034, with a *p*-value of 0.5395, and the confidence interval ranged from −0.09 to 0.16, which indicates that there is no statistically significant difference in the impact of the two groups.

**Figure 3.**
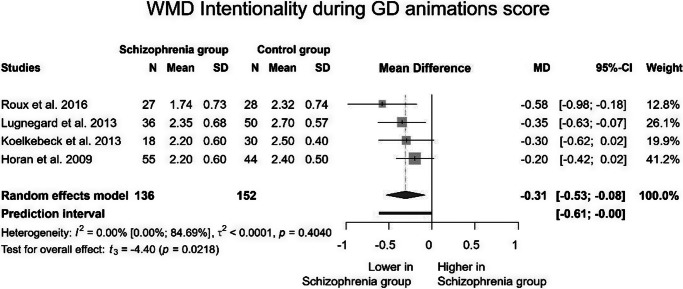
Intentionality in goal-directed animations.

For intentionality during GD animations, data from four studies (all employing the ATT paradigm) were utilized (Figure 3). These studies included 136 patients and 152 controls. A significant difference in reaction time was observed between the two groups (MD: −0.31; 95% CI (−0.53; −0.08); *p* = 0.022). Furthermore, the between-study heterogeneity, expressed as *I*^2^ value, was 0, indicating that 0% of the variance in observed effects can be attributed to true effects rather than sampling error.

### Appropriateness

**Figure 4.**
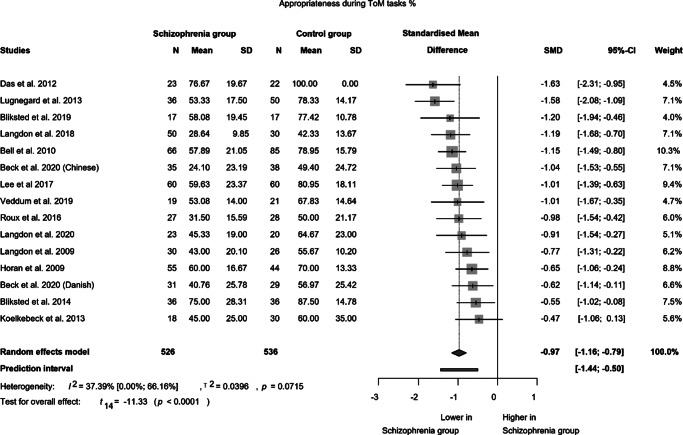
Appropriateness in mentalizing tasks.

A total of 14 studies (utilizing various methodologies) were selected for appropriateness during ToM tasks, involving a combined sample of 526 patients and 536 controls (Figure 4). A significant difference (SMD = −0.97; 95% CI (−1.16; −0.79); *p* < 0.0001) was observed between patients with schizophrenia and controls with moderate statistical heterogeneity (I^2^ = 37.4%). Schizophrenic patients exhibited a substantially weaker performance compared to controls, with an effect size of −0.97, which is regarded as a large effect.

We conducted an analysis of the appropriateness of tasks during the mentalizing tasks using the ATT study paradigm (Supplementary Figure 10). We included six studies that examined 186 patients and 190 controls. There was a significant difference between the two groups (MD: -0.63; 95% CI (−0.90; −0.35); *p* = 0.0021). The heterogeneity was considerable (I^2^ = 81.35%). The high heterogeneity may be attributed to the varying lengths of illness among the subjects. Additionally, some studies included patients with schizoaffective disorder, and most of the examined patients were also taking medication, which may have influenced the results.

A total of nine studies (utilizing diverse spontaneous mentalizing tasks) were selected for analyses of appropriateness during random tasks involving a combined sample of 274 patients and 293 controls (Supplementary Figure 11). No statistically significant difference was observed between the two groups (SMD: -0.09; 95% CI (−0.47; 0.30); *p* = 0.624). The *I*^2^ value was 68.11%, indicating substantial heterogeneity.

To assess the appropriateness during random tasks in studies using ATT, data from five studies were analysed (Supplementary Figure 12). These studies involved 163 patients and 168 controls. No significant difference was found between patients with schizophrenia and controls (MD = −0.10; 95% CI (−0.40; −0.20); *p* = 0.398). The statistical heterogeneity was substantial (*I*^2^ = 76.84%). The results are presented in Figure 9 in the supplement.

Given the outlier status of the results presented by Veddum et al. (2019)), we conducted a leave-one-out analysis by excluding this observation (Supplementary Figure 13). Despite this, no significant difference was detected between the groups (MD = −0.18; 95% CI (−0.39; 0.02); *p* = 0.0634). However, this approach resulted in a substantial reduction in heterogeneity (*I*^2^ = 20.07%).

**Figure 5.**
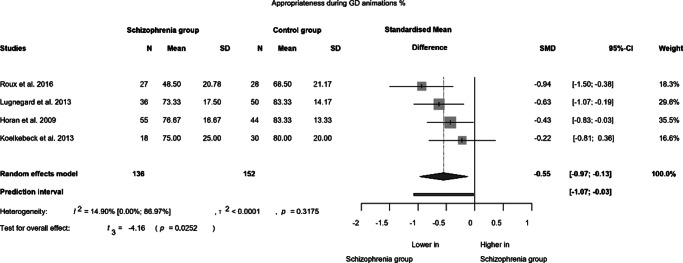
Appropriateness during goal-directed animations.

To ensure appropriateness during GD animations, data from four studies (which employed the same paradigm but utilized distinct scoring scales) were analysed. These studies involved a total of 136 patients and 152 controls. A significant difference (SMD = −0.55; 95% CI (−0.97; −0.13); *p* = 0.025) was observed between patients with schizophrenia and controls, with negligible statistical heterogeneity (*I*^2^ = 14.90%). Schizophrenic patients exhibited a weaker performance compared to controls, with an effect size of −0.55, which is classified as a medium effect. These findings are presented in Figure 5.

### Meta-regression

Meta-regression analysis was conducted for both schizophrenia and control groups, with age (in years) and time spent in education as covariates. In the control group, a significant negative correlation (*p* = 0.0492) was observed between age and appropriateness in mentalizing tasks. Similarly, a trend towards a negative correlation was observed in the schizophrenia group, although it did not reach statistical significance. No other significant effects were detected in either group. However, tendencies were observed in both groups, suggesting a potential improvement in intention during ToM tasks with increased time spent in education. Additionally, both groups exhibited lower, albeit insignificantly lower, intentionality scores during ToM tasks as a function of age.

### Assessment of the quality of evidence (GRADE Approach)

The overall assessment of the quality of evidence is presented in Table 2. The overall quality of evidence for the results was assessed as low or very low. The outcomes were downgraded due to the inconsistency and imprecision of the results. In most outcomes, the heterogeneity of the results (as determined by the *I^2^* statistic) resulted in considerable inconsistency. A substantial imprecision was caused by the relatively small sample sizes and wider confidence intervals, which further contributed to the downgrades. Regarding the risk of bias, no serious limitations were identified. In the studies included, no problems compromising the directness of the evidence were observed.

**Table 2.** Assessment of the quality of evidence

Outcome	No study/no patients/no controls	Risk of bias	Inconsistency	Indirectness	Imprecision	Quality of evidence
Intentionality during ToM	10/269/282	Not serious	Considerable	Not serious	Serious	Very Low
Intentionality during random	8/231/247	Not serious	Not serious	Not serious	Serious	Low
Intentionality during GD	4/136/152	Not serious	Some	Not serious	Serious	Low
Appropriateness during ToM %	14/526/536	Not serious	Moderate	Not serious	Serious	Low
Appropriateness during ToM	6/186/190	Not serious	Considerable	Not serious	Serious	Very Low
Appropriateness during random %	9/274/293	Not serious	Considerable	Not serious	Serious	Very Low
Appropriateness during random	5/163/168	Not serious	Considerable	Not serious	Serious	Very Low
Appropriateness during GD	4/136/152	Not serious	Not serious	Not serious	Serious	Low

### Risk of bias assessment

The overall risk of bias was low to high in the included studies. Detailed results of the quality assessment are available in the Supplementary Materials (Supplementary Figures 1–7).

## Conclusions

In our study, we reviewed the research results pertaining to spontaneous mentalization in schizophrenia. In accordance with our hypothesis, we observed impaired spontaneous mentalization skills in individuals with schizophrenia. Conversely, performance in random movement tasks did not differ significantly from that of normal control groups. However, contrary to our hypothesis, we also identified significant differences in the interpretation of GD interactions between subjects with schizophrenia and normal controls.

Patients with schizophrenia exhibited significantly lower scores in both intentionality and appropriateness in interpreting ToM interactions. Our meta-analysis revealed impaired intentionality with a large effect size, although the heterogeneity was substantial. By excluding one outlying study, the heterogeneity decreased considerably, but the difference between the groups remained significant. Furthermore, significant differences were also observed between the groups in appropriateness with a large effect size and low heterogeneity. These findings suggest a substantial deficit in spontaneous ToM in schizophrenia, particularly evident in patients who performed equally to healthy controls in the control condition with random animations. These results imply that patients with schizophrenia are less likely to appropriately capture situations requiring spontaneous mentalizing activity and exhibit a lower degree of mental state attribution in describing the interactions.

Effect sizes (intentionality: −0.99, appropriateness: −0.97) in our meta-analysis indicate that the severity of impairment in spontaneous mentalizing is comparable to the level of impairment reported in explicit mentalizing meta-analyses (e.g., Bora et al., 2009; Bora & Pantelis, 2013). Furthermore, we observed significantly longer reaction times with a comparable effect size (0.89) in our previous meta-analysis on implicit ToM (Csulak et al., 2022). Reaction time serves as a proxy for the interference effect of intention detection, making it a suitable measure to assess implicit ToM in indirect task paradigms (Gardner & Buchanan, 2023). Overall, the meta-analyses conducted on various aspects of ToM skills in schizophrenia reveal a pervasive deficit in mentalizing abilities.

Additionally, meta-regression revealed a negative correlation between age and mentalizing performance in control subjects, which aligns with the findings suggesting that older adults tend to experience a decline in mentalizing abilities (Cassidy, Hughes, & Krendl, 2021; Greenberg, Warrier, Abu-Akel, & Baron-Cohen, 2023). A similar trend was observed in patients, although the results were not statistically significant.

Patients also performed significantly worse in GD conditions, although the effect sizes were lower in detecting and describing GD animations compared to the mentalizing condition. However, due to limited data, we could not analyze the relationship between mentalizing and goal-oriented animations. Only four studies were included from the available seven due to data limitations, reducing the generalizability of the findings. The lower performance in the GD animation condition suggests a deeper deficit, potentially indicative of general cognitive dysfunction. Pedersen et al. (2012)) emphasized the significance of reaction time in understanding social cognition. In their functional imaging study, similar brain areas were activated in patients and control individuals during the observation of triangles, but the activation occurred later in the patients. Given that the processing of ATT stimuli entails an implicit mentalizing component (Bliksted et al., 2016), we can also posit that cognitive impairments may hinder the performance in ATT through the implicit processing. Furthermore, data on implicit mentalizing suggest that impaired neurocognition (e.g., reaction time) may play a limiting role in implicit processing in schizophrenia (Csulak et al., 2022). Lower performance in GD and ToM animations may indicate impaired visual processing of moving objects. The processing of biological motion by living organisms is crucial to social cognition, including mentalizing (Kim et al., 2013; Pavlova, 2012). A meta-analysis found that patients with schizophrenia perform poorly on basic biological motion tasks and emotion recognition tasks (Okruszek & Pilecka, 2017). However, the equal performance in random tasks in our study contradicts this assumption. It seems a more plausible explanation that GD and mentalizing tasks require more cognitive effort than random tasks. The lower performance in GD may also reflect an inappropriate mentalizing activity. A study conducted by Russell, Reynaud, Herba, Morris, and Corcoran (2006)) revealed that patients provided more mentalizing responses in the GD condition compared to controls. This raises the question of whether the GD condition accurately represents a GD interaction without the mentalizing aspect. GD task can also be interpreted as a fundamental mentalizing task (Roux et al., 2016). These uncertainties led to a recent meta-analysis of autism spectrum research underscoring the low-level construct validity of ATT (Wilson, 2021).

The lower performance in schizophrenia may indicate patients’ restricted ability to assign narratives to moving stimuli. Linguistic research suggests that narrative language production problems in schizophrenia are linked to semantic deficits and cognitive impairments, resulting in less coherent and clear spoken life narratives (Marini & Perlini, 2013, Lundin, Cowan, Singh, & Moe, 2023). They even exhibit impaired understanding of the narrative content of literary fictions and use fewer mental state terms spontaneously to describe social events (Fekete et al., 2020). Narrative processing requires synchronized cooperation of mentalizing, language, and neurocognitive skills (Delgado et al., 2024). Longitudinal studies reveal the interplay between these abilities (Ebert, 2020; Shahaeian, Haynes, & Frick, 2023). Spontaneous mentalizing performance also correlated with reasoning, verbal IQ, and verbal memory in schizophrenia (Koelkebeck et al., 2010). Roux et al. (2016)) and Koelkebeck et al. (2013)) employed a scoring scale to measure utterances during the ATT task. Patients’ descriptions of tasks were comparable to controls’, but they used fewer intentional and action-describing terms, suggesting repetitions and descriptions of physical reality rather than actions. Majority of the research indicates that patients with schizophrenia also tend to provide shorter responses and use mental state terms less frequently (Andreasen et al. 2008; Bliksted et al., 2016; Langdon, Connors et al., 2017). Furthermore, some studies have suggested a weak correlation between the WAIS Vocabulary test and schizophrenia (Bell et al., 2010; Lugnegård et al., 2013). Consequently, we propose that vocabulary, including mental state terms, may also be compromised in schizophrenia. Recent longitudinal studies emphasize the role of vocabulary learning in mentalizing development (Delgado, 2024; Ebert, 2020). Regrettably, we were unable to analyze the results on verbal performance during the spontaneous mentalizing tasks due to data incompatibility difficulties. In this regard, it would have been beneficial if sufficient data had been available to assess the impact of general intelligence. Some studies lacked IQ measurement (Bell et al., 2010; Das et al., 2012; Koelkebeck et al., 2013), conducted only subtests (Horan et al., 2009; Lugnegård et al., 2013; Pedersen et al., 2012; Veddum et al., 2019; Beck et al., 2020), or measured premorbid estimated intelligence levels (Langdon et al., 2020; Langdon & Ward, 2009). Studies that measured IQ yielded conflicting results. Some found a correlation with spontaneous mentalization (Bliksted et al., 2014; Lee et al., 2018), while others concluded that IQ does not account for diminished performance in schizophrenia (Roux et al., 2016).

There was no adequate data for meta-regression in the case of symptomatology either. Some studies found no significant relationship between symptoms and mental performance (Bliksted et al., 2014; Das et al., 2012; Langdon, 2017; Roux et al., 2016; Bell et al., 2010; Lee et al., 2018). Others described correlations with negative symptoms (Horan et al., 2009), referential ideation, but not with persecutory ideation (Langdon et al., 2020), or with the higher levels of both positive and negative symptoms (Bliksted et al., 2016). Interestingly, Bliksted et al. (2019) described different neural activation during intentional scenes depending on symptoms. However, not all studies reported direct data on the correlations of symptoms and mentalizing.

In summary, individuals with schizophrenia exhibit challenges in spontaneously activating their mentalizing capacity in social contexts. This highlights the significance of enhancing spontaneous mentalization skills. However, as schizophrenia is characterized by a pervasive mentalization deficit, interventions providing compensatory strategies for explicit mentalization are necessary, but supporting spontaneous mentalization is also crucial. Meta-analytic evidence supports the utility of broad-based social cognition training in enhancing explicit mentalizing abilities in individuals with schizophrenia, though its durability remains uncertain (Nijman, Veling, van der Stouwe, & Pijnenborg, 2020). However, limited evidence exists for improving spontaneous mentalization. Attention training (Langdon, Connors et al., 2017) or imitation–inhibition training that facilitates perspective-taking abilities may be considered as a potential approach (Simonsen et al., 2020). Enhancing mental state vocabulary, for instance, through reading literary fiction, could also contribute to improved spontaneous mentalization performance (Fekete et al., 2023). Mentalization-based therapy offers a novel approach, but its efficacy in schizophrenia spectrum disorders is still uncertain. Studies suggest it helps patients with schizophrenia understand social causality (Weijers et al., 2023) and may benefit recent-onset patients (Weijers et al., 2021).

### Limitations

Our meta-analysis has several limitations, the primary one being the relatively low number of eligible studies. Regrettably, several studies were excluded due to insufficient data for meta-analysis. However, it is important to note that, according to the Cochrane Handbook for Systematic Reviews, a meta-analysis is the statistical combination of results from two or more separate studies. Furthermore, as asserted by Valentine, Pigott, and Rothstein (2010)), at least two studies are necessary for a meta-analysis because it remains the most transparent and valid method of synthesizing research data. Additionally, there was heterogeneity in the application of research paradigms. Among the 15 studies included in the meta-analysis, various approaches were employed to assess ToM: 10 studies utilized the ATT, three articles employed the joke appreciation task, and two utilized the SAT-MC. Moreover, not all aspects of the original task were consistently utilized across different research studies. For instance, not all studies of ATT employed the three types of tasks (random, GD, and mentalizing). The heterogeneity of the research designs employed significantly restricts the generalizability of our findings. This limitation is somewhat mitigated by the fact that the analysis of data employing solely the ATT paradigm yielded comparable results. Heterogeneity was also apparent in the sample selection. In most studies, the average duration of illness was up to 10 years, while others included first-episode patients. Another limitation of our study pertains to meta-regression analysis. Due to data incompatibility (utilization of distinct testing tools), we were unable to examine the association between mentalizing performance during ToM tasks and IQ, nor the presence of symptoms, as data from at least 10 studies are needed to perform meta-regression (Higgins & Green, 2011). Both dimensions are crucial mediators of mentalizing performance. For instance, higher verbal intelligence is associated with enhanced activation of brain regions involved in mentalization (Tantchik et al., 2023), while lower intelligence is linked to more pronounced deficits in mentalizing tasks (Sahl et al., 2022).

In conclusion, this meta-analysis shows that schizophrenia patients have significant impairments in spontaneous mentalizing. They have reduced sensitivity to bottom-up signals that trigger mental state attributions and diminished intentionality and appropriateness in describing social interactions (Langdon, Connors et al., 2017). These impairments disrupt the automatic mobilization of mentalizing skills, which are crucial for effective social functioning. While performance deficits were pronounced in mentalizing tasks, patients showed no significant differences in random animation tasks. However, they had impairments in GD tasks, suggesting a more generalized cognitive dysfunction. The findings also highlight the pervasive nature of mentalizing deficits, encompassing explicit, implicit, and spontaneous dimensions. These findings have implications for developing tailored remediation strategies to enhance social cognition and functional outcomes for individuals with schizophrenia.

## Supporting information

Hajnal et al. supplementary material Hajnal et al. supplementary material

## Supplementary material

The supplementary material for this article can be found at http://doi.org/10.1017/S0033291725100755.

## Appendix. Paradigms for assessing spontaneous mentalizing

ATT: During the ATT (Abell et al., 2000), participants observe 34–45 second animations depicting the movements and interactions of a large and a small red triangle. These animations are categorized into three types: random movements (e.g., bouncing), goal-oriented movements (e.g., chasing), and animations that incorporate mentalizations (e.g., surprising, mocking). Participants’ responses are evaluated based on several criteria. These include the extent to which their appreciation of mental states (intentionality) is evident, the accuracy of the animations’ description (appropriateness), and, in some trials, the length of their responses.

Heider and Simmel’s Animation: Langdon, Connors et al. (2017) utilized the Heider and Simmel’s animation, which incorporates the social interaction of geometric shapes (two triangles and a circle). This test elicits intentionality and emotional mental status. Inspired by Klin’s (2000) study, participants were asked, ‘What happened there?’ Subsequently, a cue instruction was applied, personifying geometric shapes without explicitly representing them as intentional agents. During the test, patients were evaluated for spontaneous responses, total word count, accuracy of basic and social expressions, and the utilization of various mental state expressions (perception, emotion, goal-driven intentions).

Social Attribution Task – Multiple Choice version (SAT-MC): During the SAT-MC examination (Bell et al., 2010), participants are presented with a 64-second animation (Heider and Simmel’s animation) depicting a social interaction involving two triangles and a circle. This recording is viewed twice. Subsequently, 19 questions are posed, requiring participants to select the correct answer from four possible options.

Joke Appreciation Task (Langdon & Ward, 2009): Two sets of cartoons are presented, one of which necessitates mentalizing, and the other does not. Participants are then requested to elucidate the joke, and their responses are subsequently evaluated on a three-point scale.
