# Supplementary figures and images for: Spontaneous mentalizing in patients with schizophrenia spectrum disorders: a meta-analysis
Source: Psychol Med. 2025 Jul 16;55:e195. doi: 10.1017/S0033291725100755 (PMC12315645; doi:10.1017/S0033291725100755)

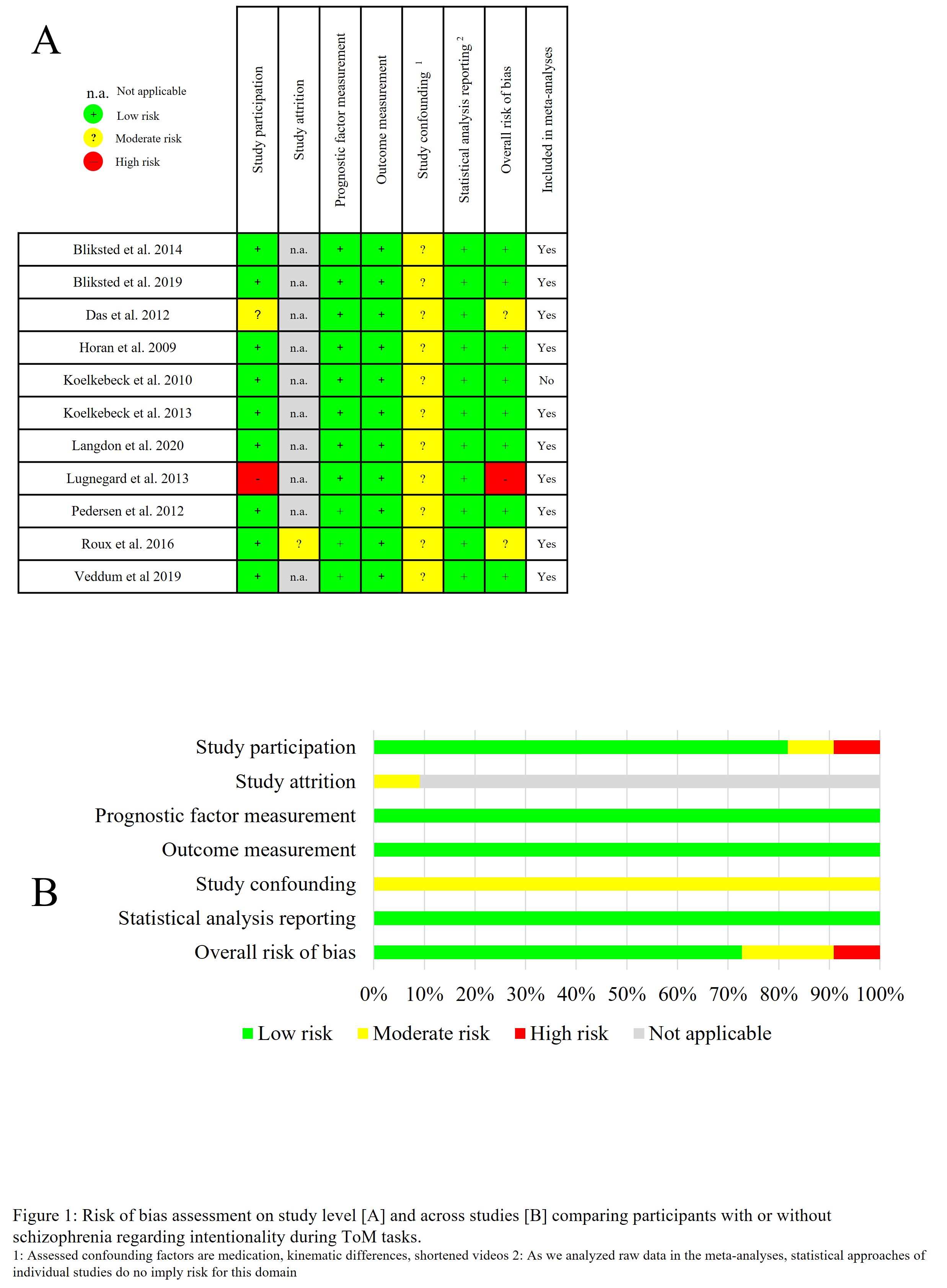

Supplement: Hajnal et al. supplementary material [file S0033291725100755sup001.zip › SF 1.jpg]

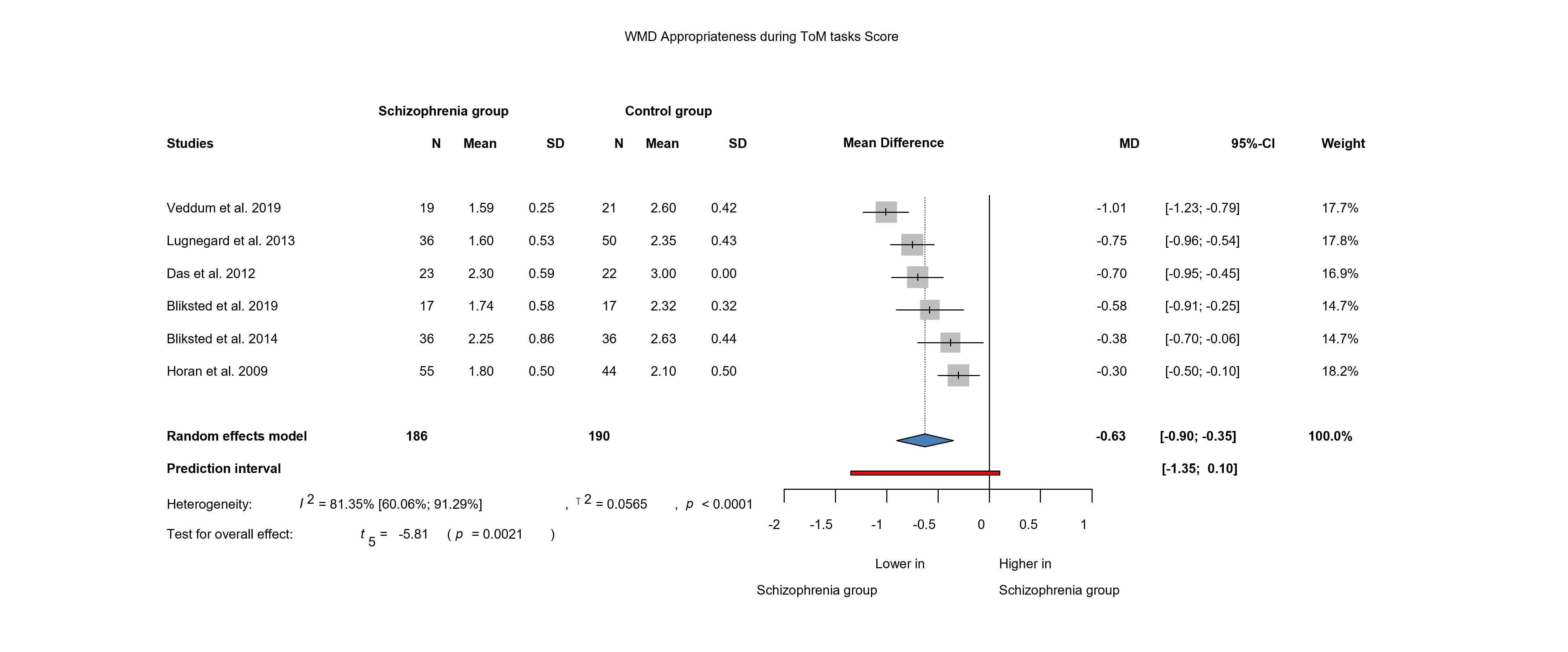

Supplement: Hajnal et al. supplementary material [file S0033291725100755sup001.zip › SF 10.tif]

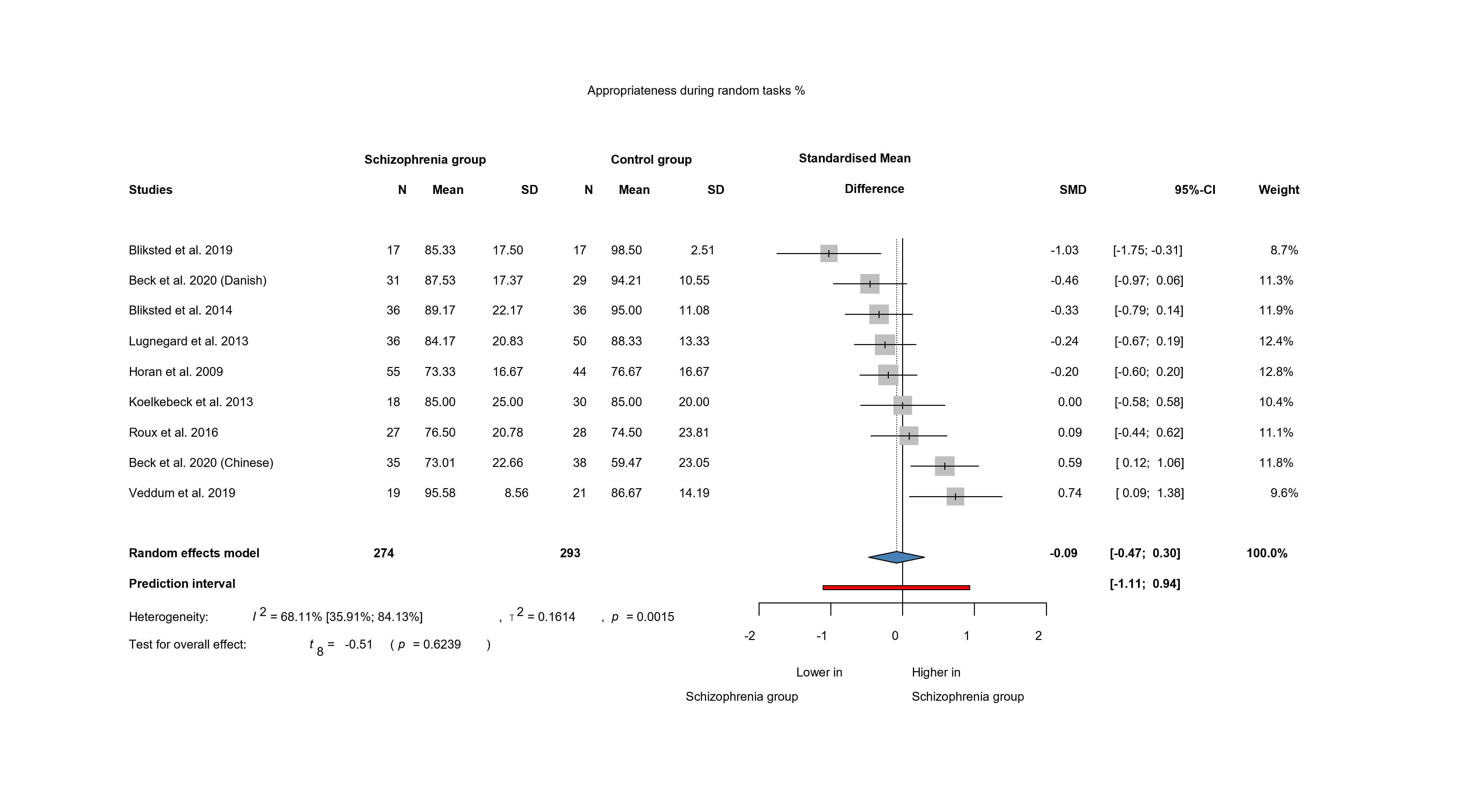

Supplement: Hajnal et al. supplementary material [file S0033291725100755sup001.zip › SF 11.tif]

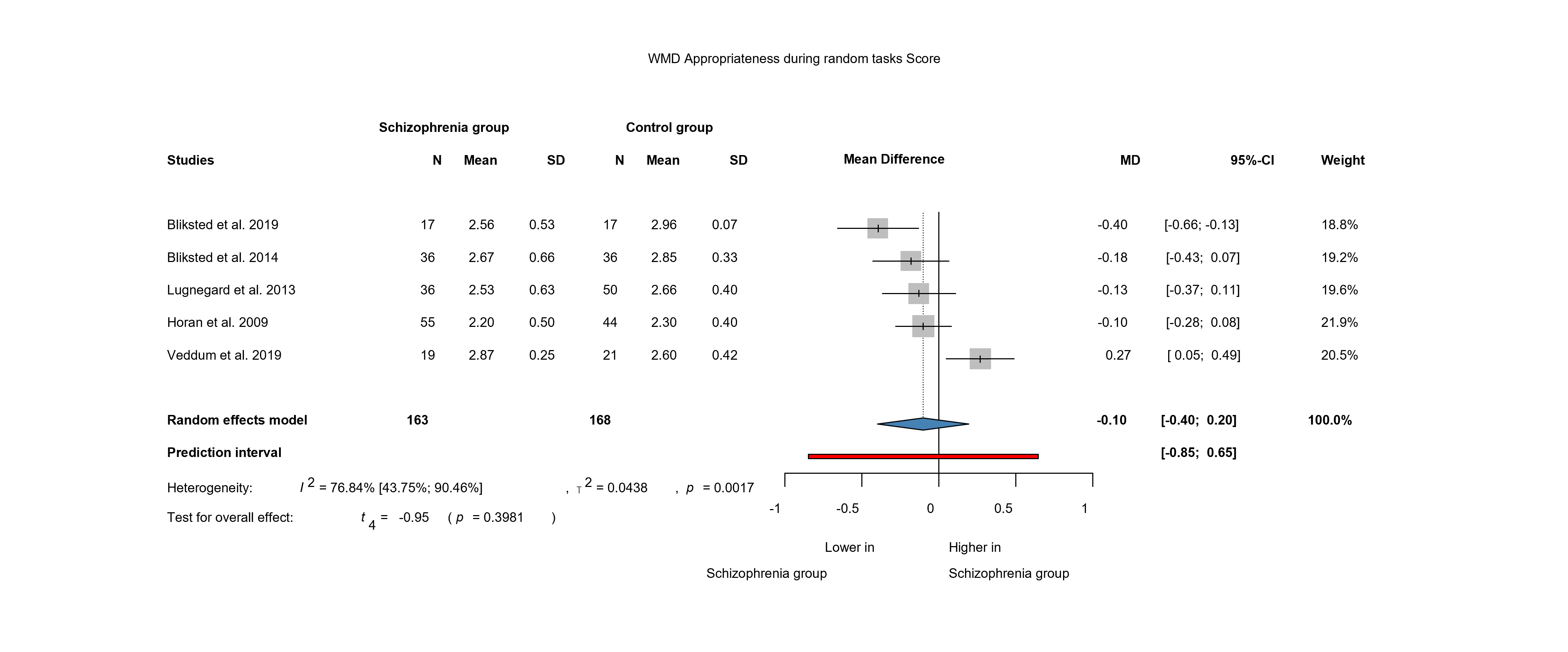

Supplement: Hajnal et al. supplementary material [file S0033291725100755sup001.zip › SF 12.tif]

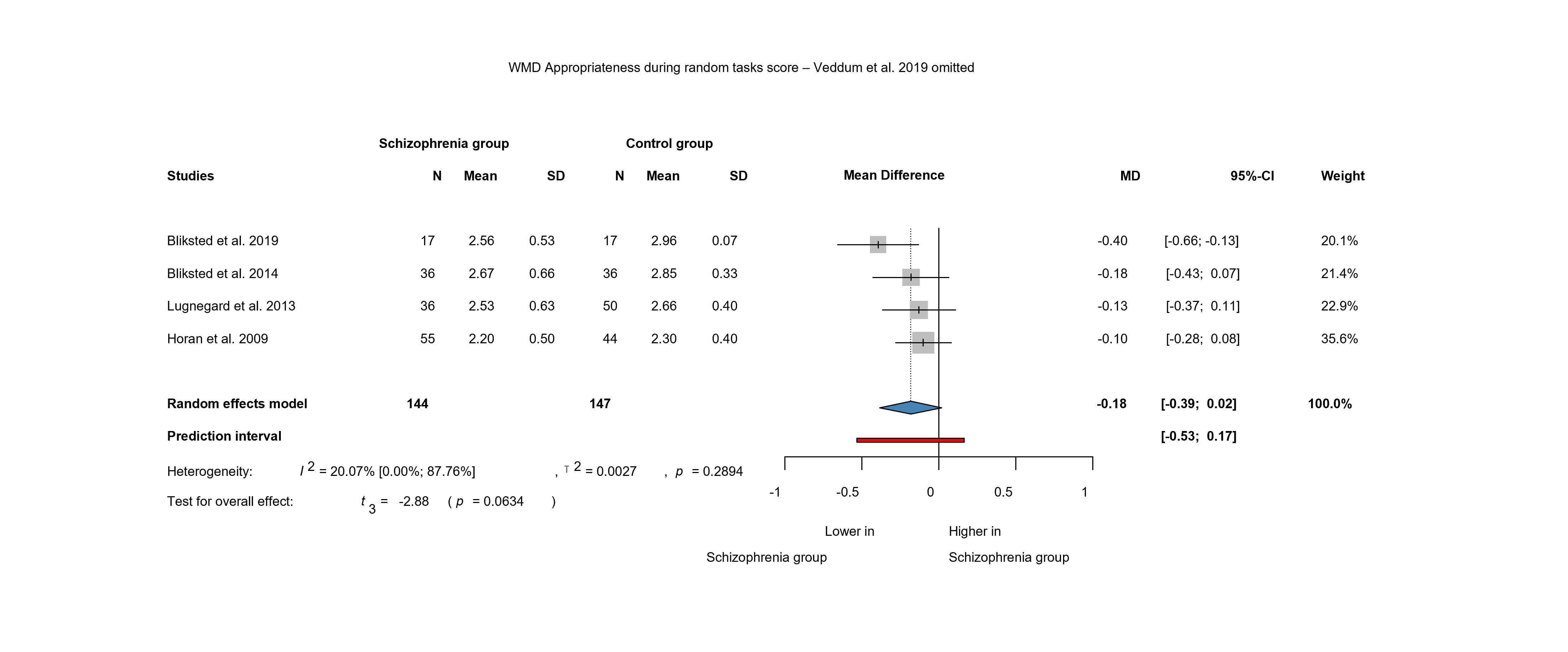

Supplement: Hajnal et al. supplementary material [file S0033291725100755sup001.zip › SF 13.tif]

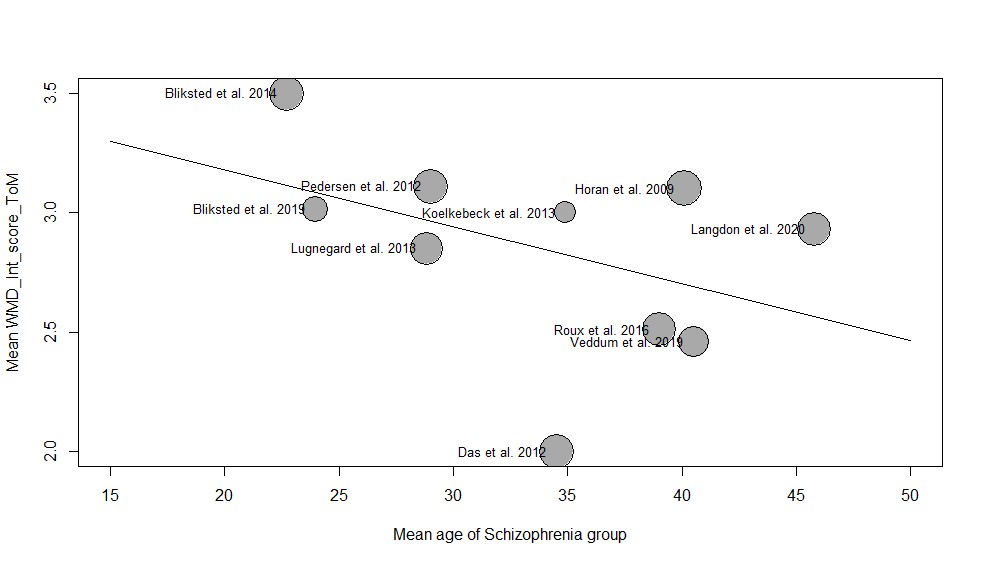

Supplement: Hajnal et al. supplementary material [file S0033291725100755sup001.zip › SF 14.jpg]

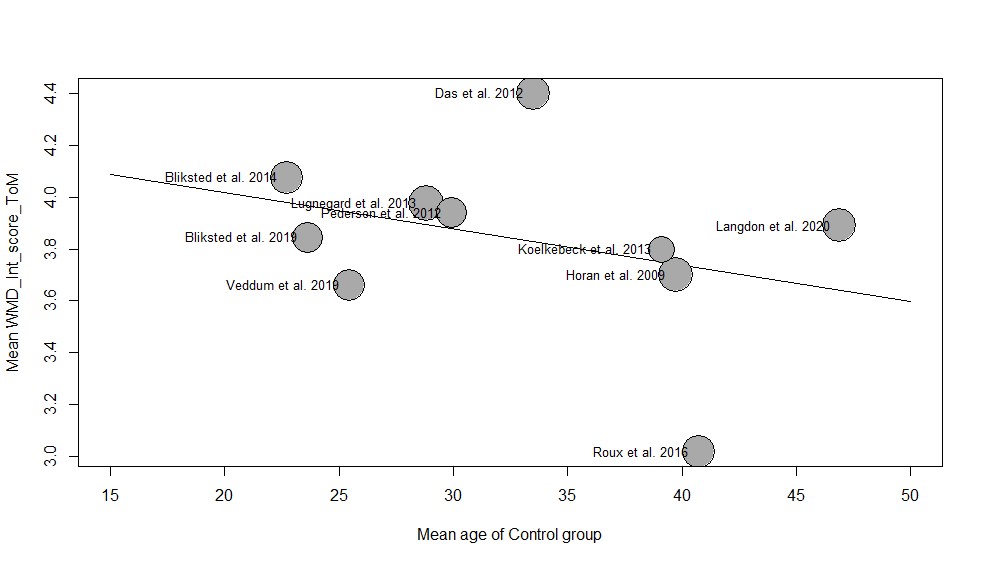

Supplement: Hajnal et al. supplementary material [file S0033291725100755sup001.zip › SF 15.jpg]

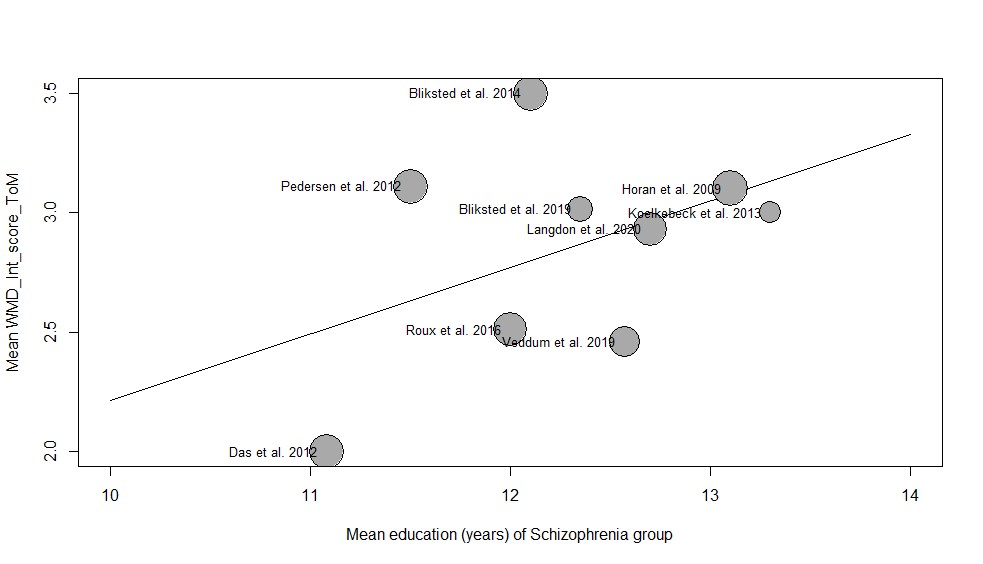

Supplement: Hajnal et al. supplementary material [file S0033291725100755sup001.zip › SF 16 .jpg]

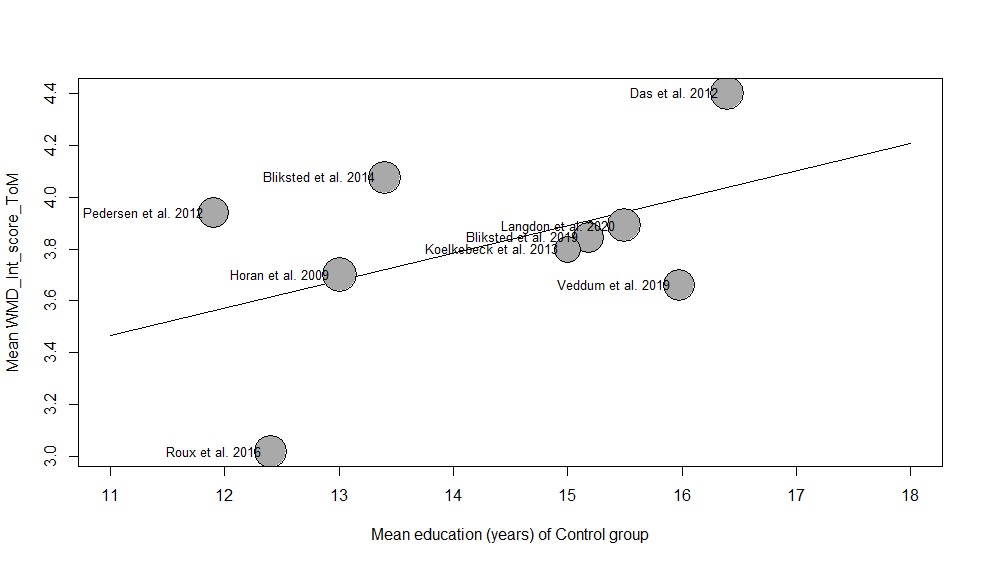

Supplement: Hajnal et al. supplementary material [file S0033291725100755sup001.zip › SF 17.jpg]

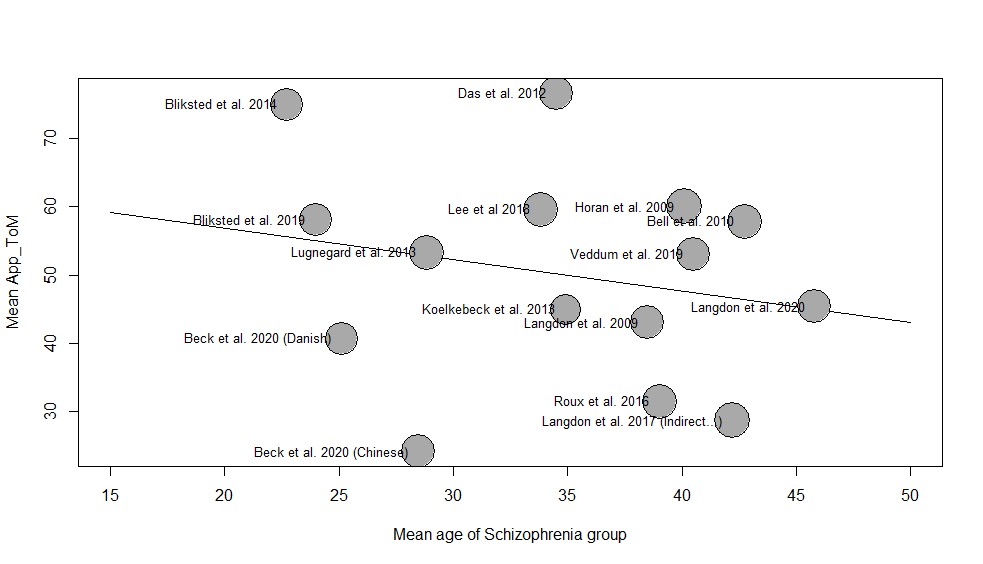

Supplement: Hajnal et al. supplementary material [file S0033291725100755sup001.zip › SF 18.jpg]

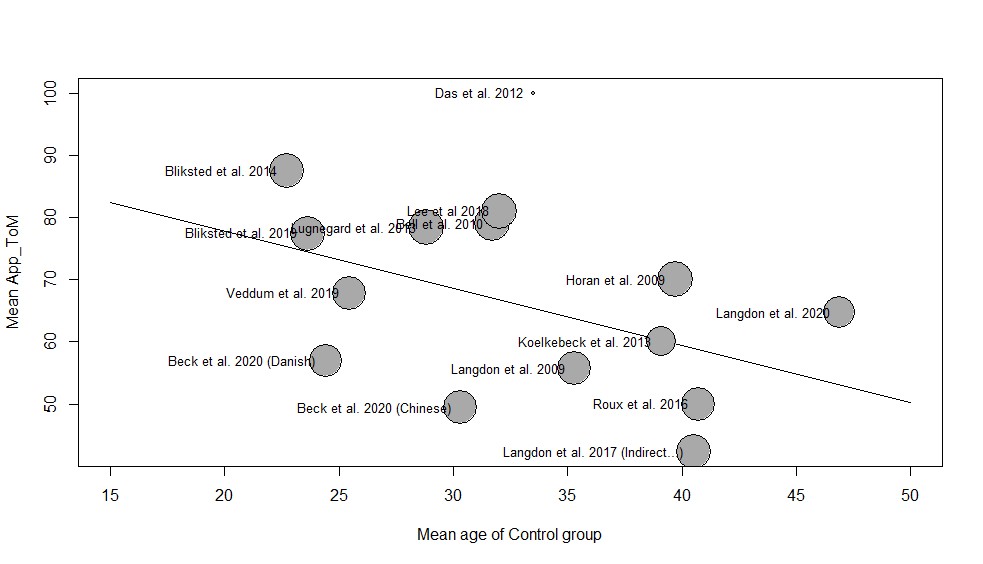

Supplement: Hajnal et al. supplementary material [file S0033291725100755sup001.zip › SF 19.jpg]

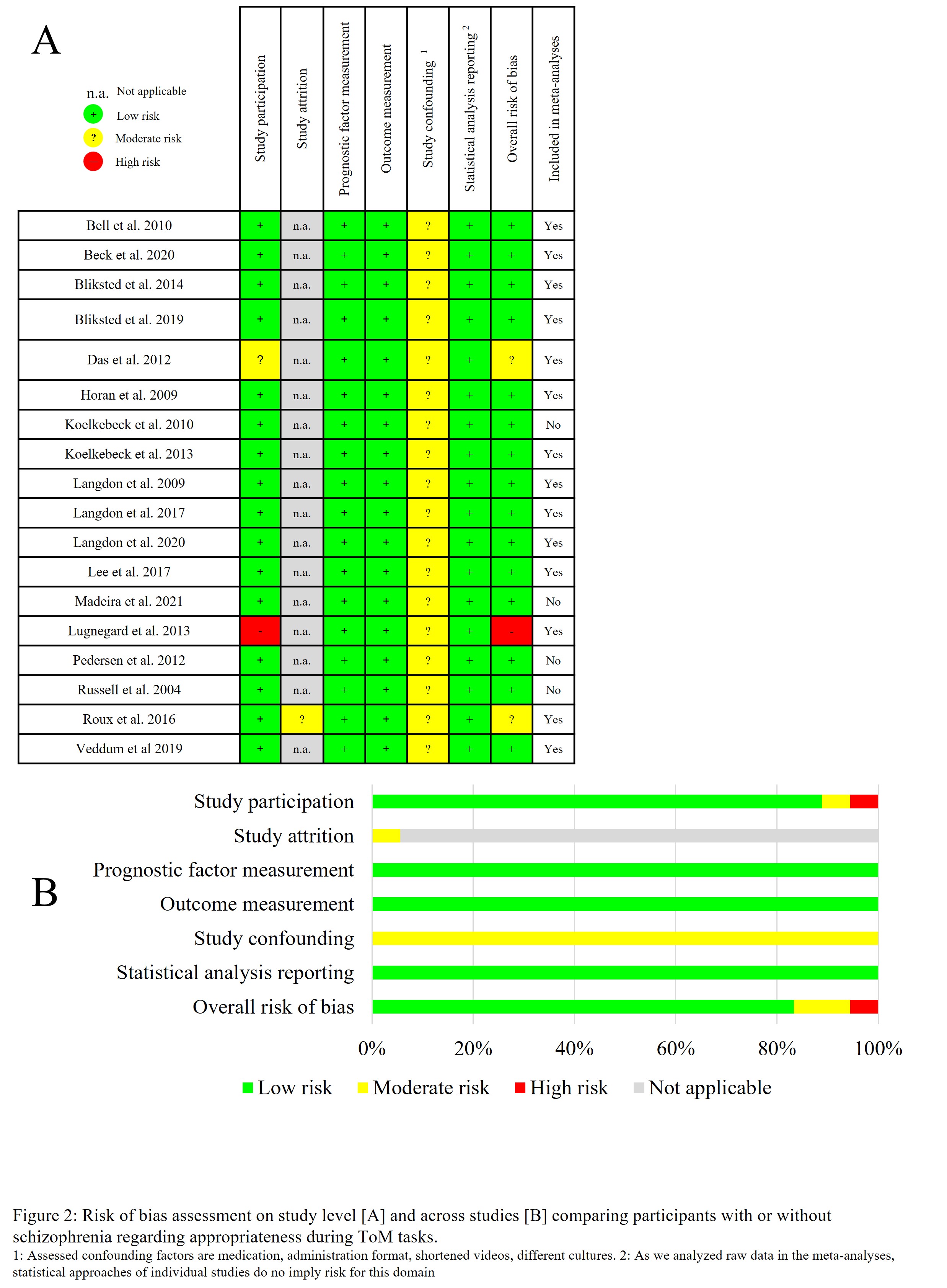

Supplement: Hajnal et al. supplementary material [file S0033291725100755sup001.zip › SF 2.jpg]

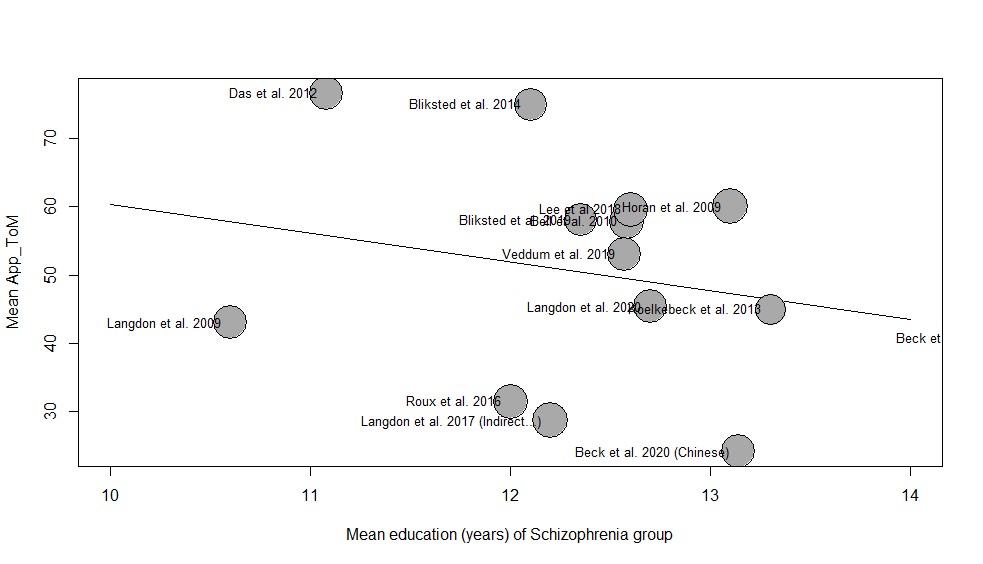

Supplement: Hajnal et al. supplementary material [file S0033291725100755sup001.zip › SF 20.jpg]

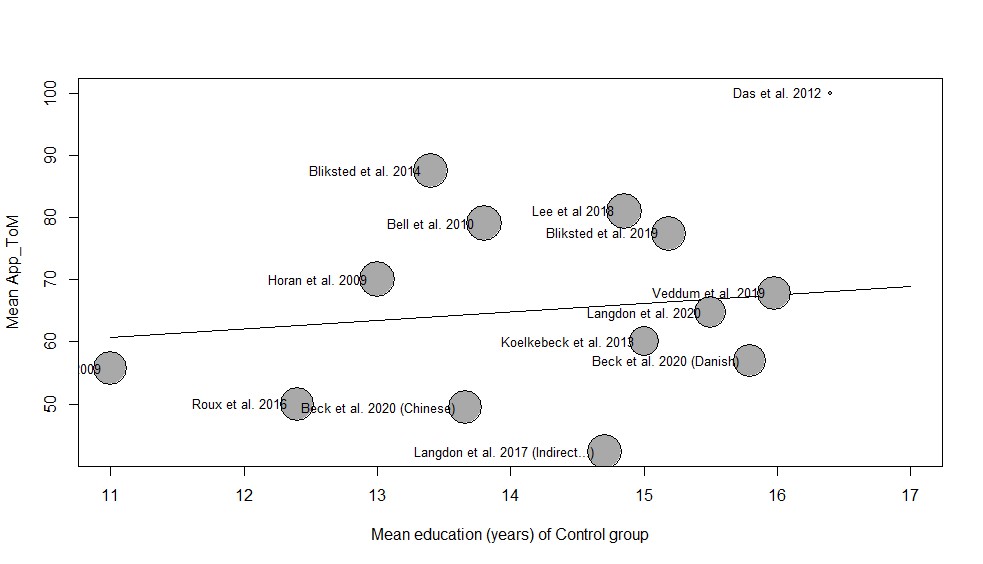

Supplement: Hajnal et al. supplementary material [file S0033291725100755sup001.zip › SF 21.jpg]

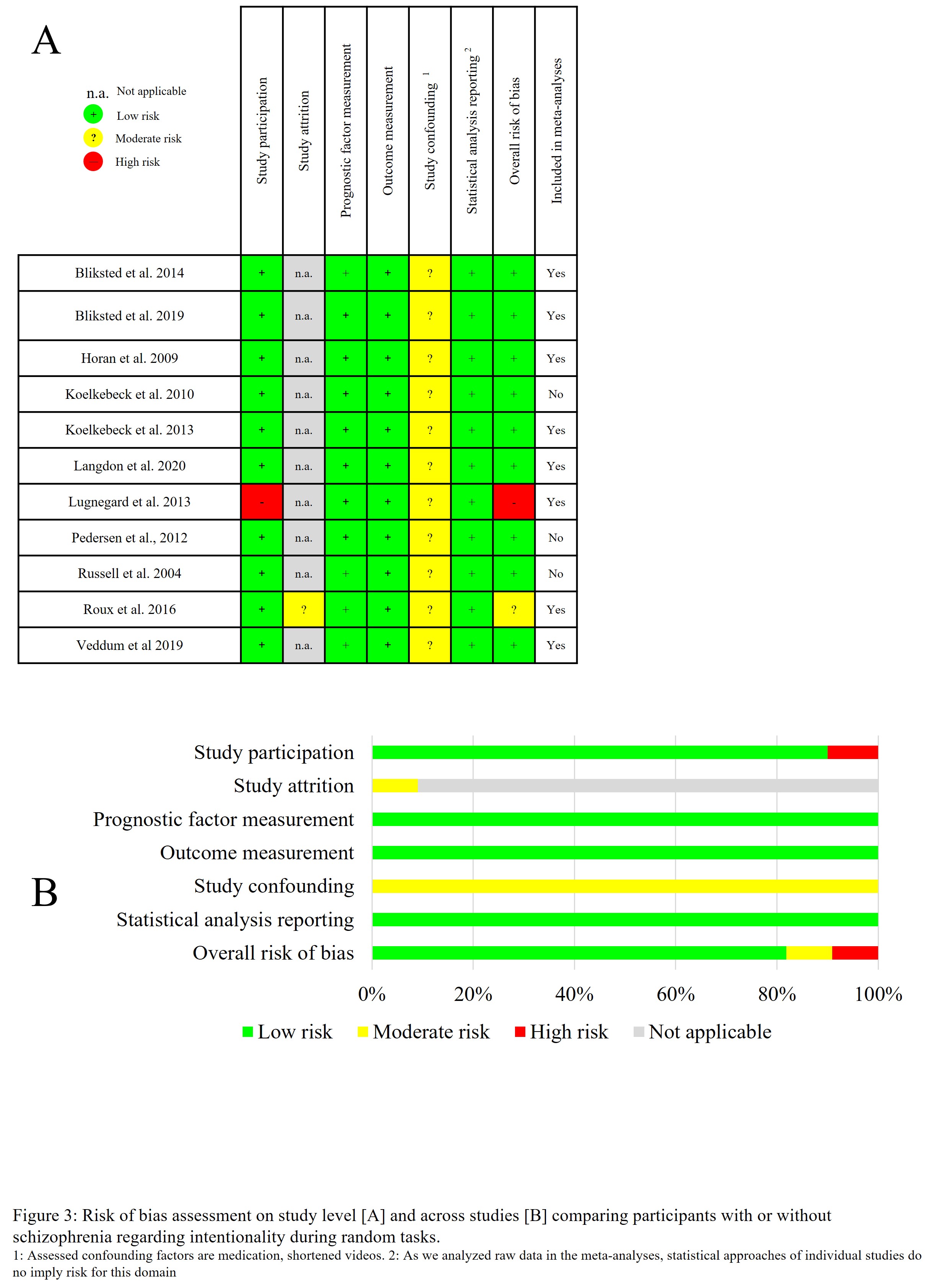

Supplement: Hajnal et al. supplementary material [file S0033291725100755sup001.zip › SF 3.jpg]

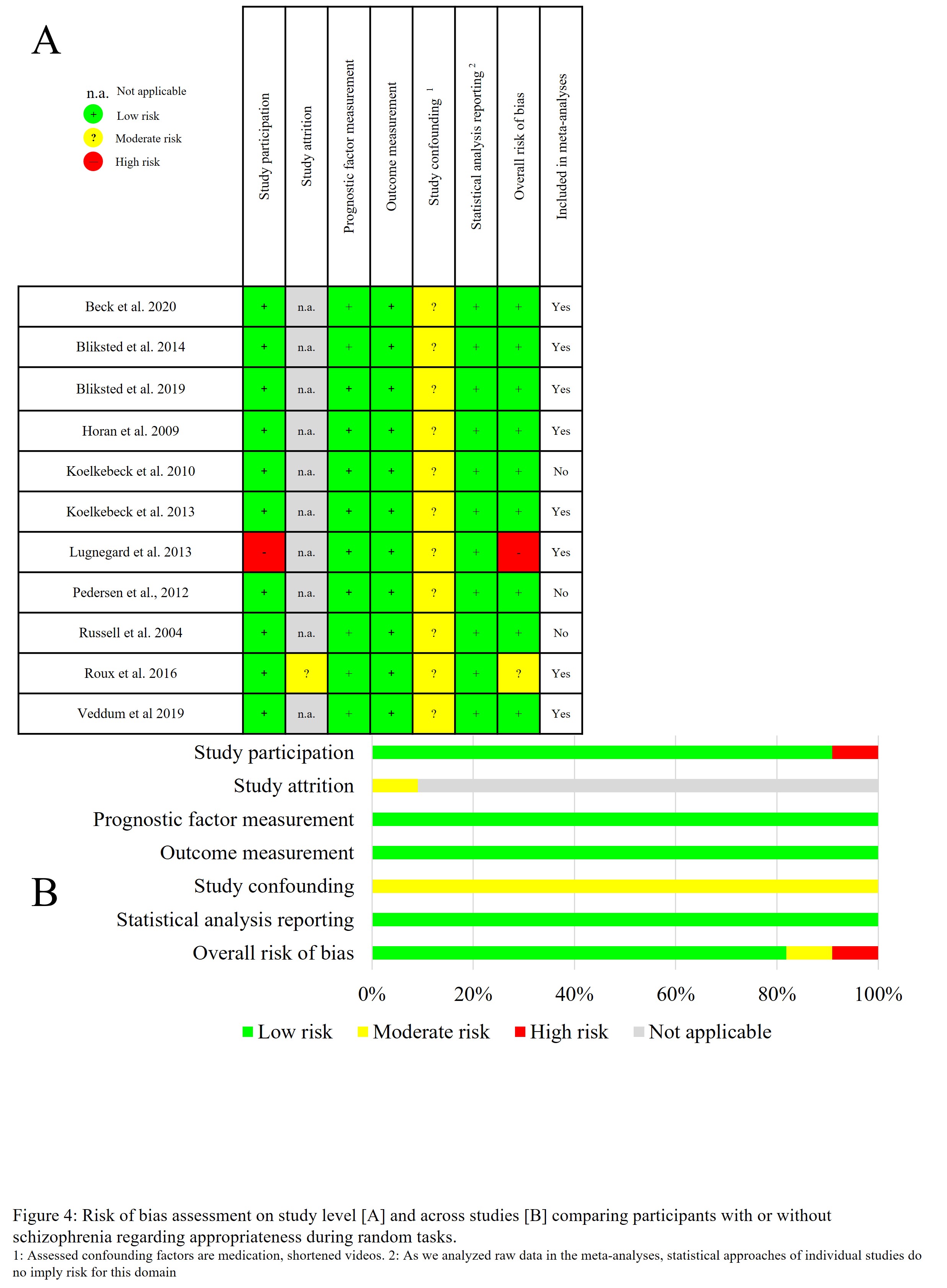

Supplement: Hajnal et al. supplementary material [file S0033291725100755sup001.zip › SF 4.jpg]

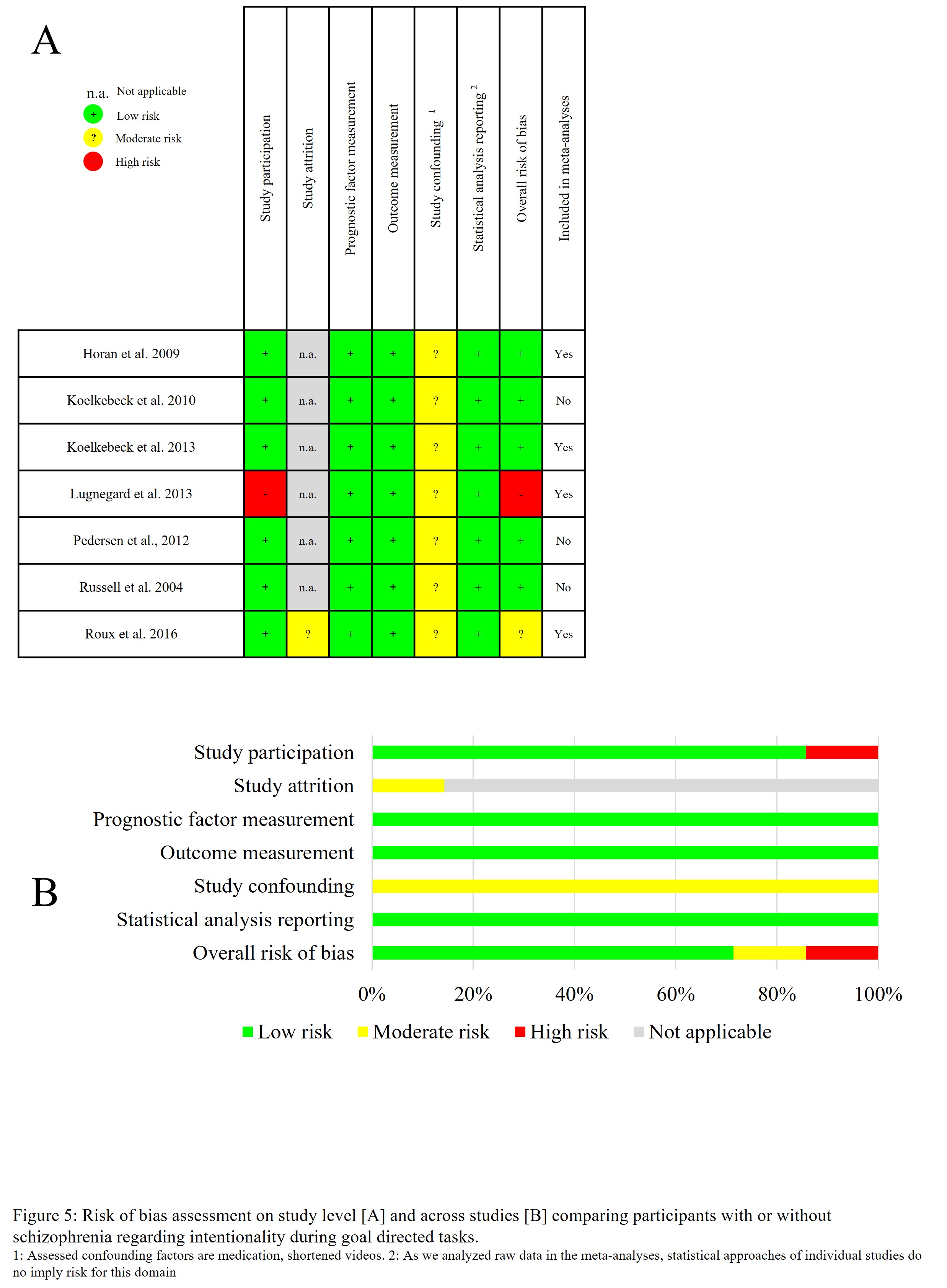

Supplement: Hajnal et al. supplementary material [file S0033291725100755sup001.zip › SF 5.jpg]

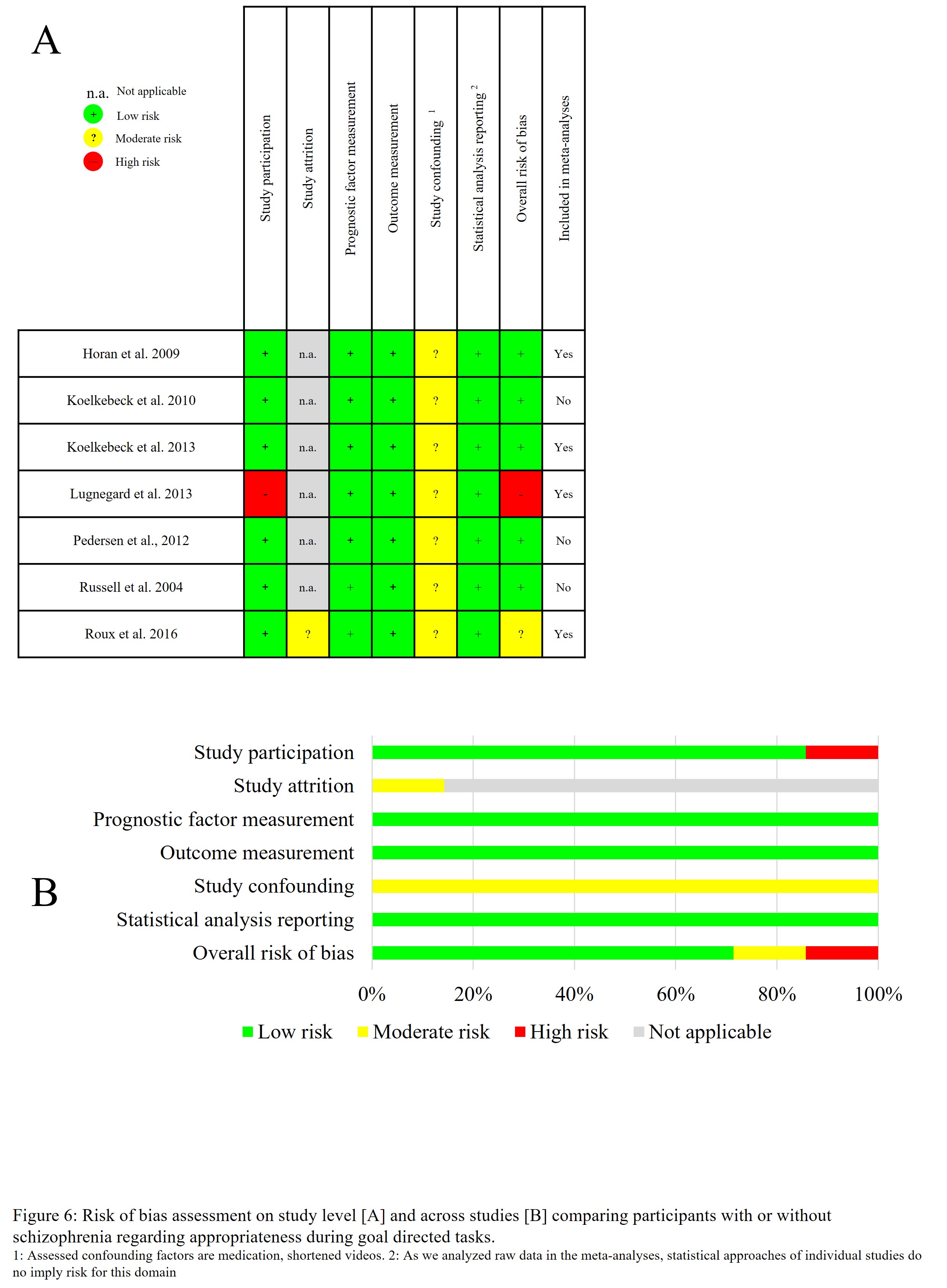

Supplement: Hajnal et al. supplementary material [file S0033291725100755sup001.zip › SF 6.jpg]

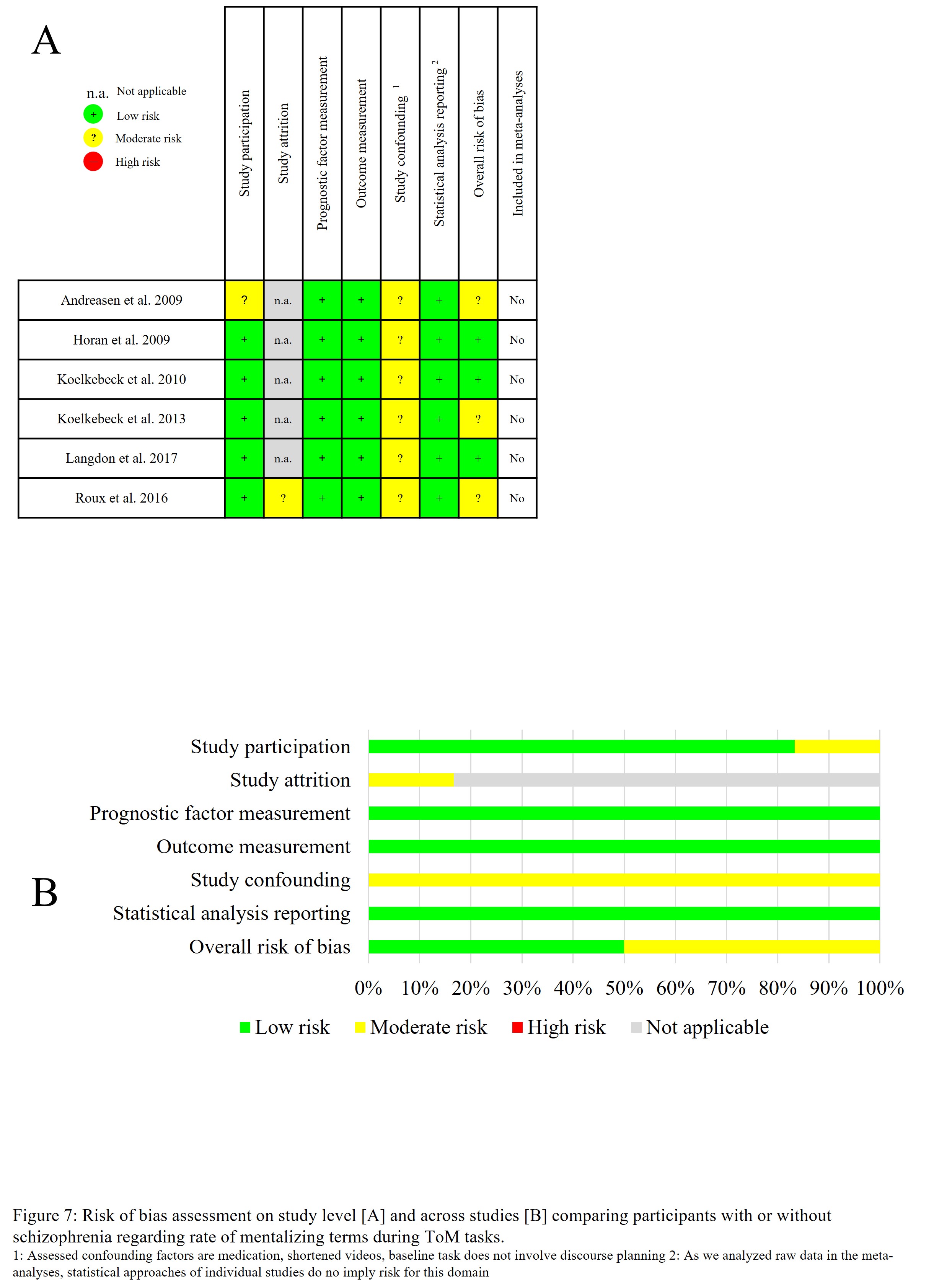

Supplement: Hajnal et al. supplementary material [file S0033291725100755sup001.zip › SF 7.jpg]

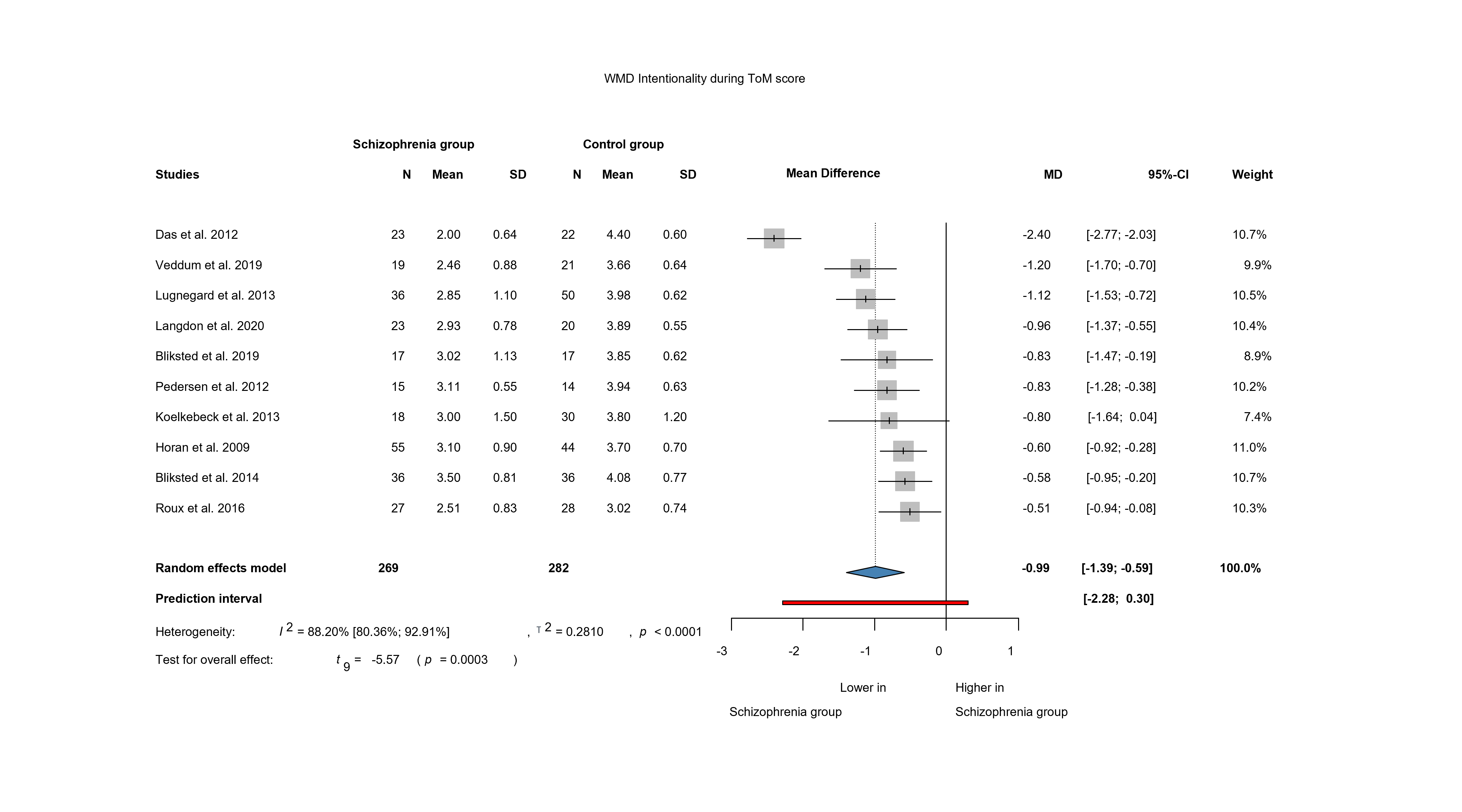

Supplement: Hajnal et al. supplementary material [file S0033291725100755sup001.zip › SF 8.tif]

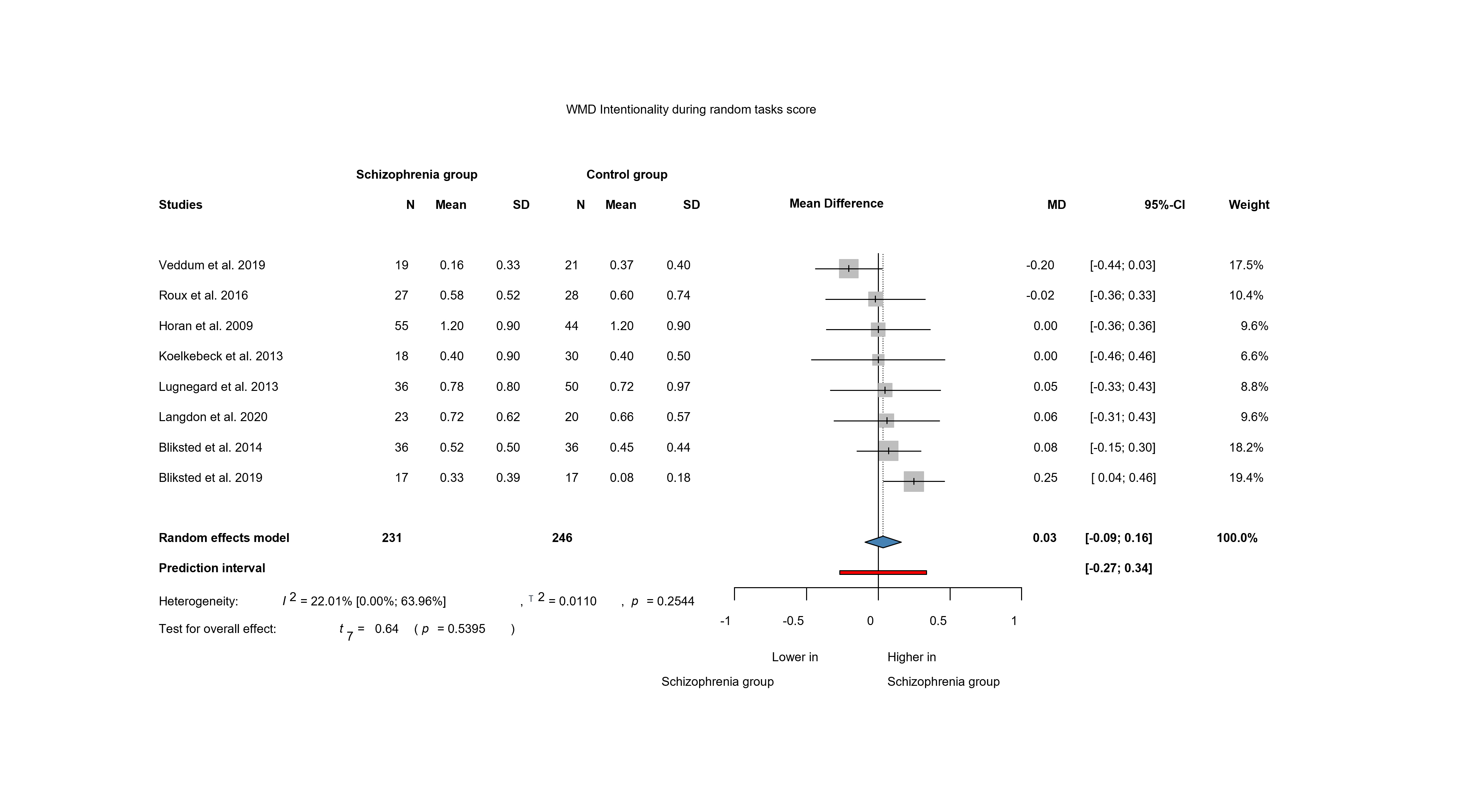

Supplement: Hajnal et al. supplementary material [file S0033291725100755sup001.zip › SF 9.tif]
